# Isolation and Functional Characterization of the Promoters of Miltiradiene Synthase Genes, *TwTPS27a* and *TwTPS27b*, and Interaction Analysis with the Transcription Factor TwTGA1 from *Tripterygium wilfordii*

**DOI:** 10.3390/plants10020418

**Published:** 2021-02-23

**Authors:** Yanbo Huo, Bin Zhang, Ling Chen, Jing Zhang, Xing Zhang, Chuanshu Zhu

**Affiliations:** 1 College of Plant Protection, Northwest A&F University, Yangling 712100, China; huoyanbo@nwsuaf.edu.cn (Y.H.); zhangbin1990@nwsuaf.edu.cn (B.Z.); Chenling@nwsuaf.edu.cn (L.C.); zhjing008@nwsuaf.edu.cn (J.Z.); 2 Engineering and Research Center of Biological Pesticide of Shaanxi Province, Yangling 712100, China

**Keywords:** promoter analysis, TGACG-motif, MeJA-responsive, binding site, interaction, regulatory mechanism, triptolide

## Abstract

Miltiradiene synthase (MS) genes, *TwTPS27a* and *TwTPS27b*, are the key diterpene synthase genes in the biosynthesis of triptolide, which is an important medicinally active diterpenoid in *Tripterygium wilfordii*. However, the mechanism underlying the regulation of key genes *TwTPS27a/b* in triptolide biosynthesis remains unclear. In this study, the promoters of *TwTPS27a* (1496 bp) and *TwTPS27b* (1862 bp) were isolated and analyzed. Some hormone-/stress-responsive elements and transcription factor (TF) binding sites were predicted in both promoters, which might be responsible for the regulation mechanism of *TwTPS27a/b*. The β-glucuronidase (GUS) activity analysis in promoter deletion assays under normal and methyl jasmonate (MeJA) conditions showed that the sequence of −921 to −391 bp is the potential core region of the *TwTPS27b* promoter. And the TGACG-motif, a MeJA-responsive element found in this core region, might be responsible for MeJA-mediated stress induction of GUS activity. Moreover, the TGACG-motif is also known as the TGA TF-binding site. Yeast one-hybrid and GUS transactivation assays confirmed the interaction between the *TwTPS27a/b* promoters and the TwTGA1 TF (a MeJA-inducible TGA TF upregulating triptolide biosynthesis in *T. wilfordii*), indicating that *TwTPS27a/b* are two target genes regulated by TwTGA1. In conclusion, our results provide important information for elucidating the regulatory mechanism of MS genes, *TwTPS27a* and *TwTPS27b*, as two target genes of TwTGA1, in jasmonic acid (JA)-inducible triptolide biosynthesis.

## 1. Introduction

*Tripterygium wilfordii* is a well-known traditional medicinal herb whose common name is Lei Gong Teng or thunder duke vine [1,2]. The identification of hundreds of effective compounds isolated from the roots of *T wilfordii* [3,4], such as diterpenoids, triterpenoids, sesquiterpenoids, and alkaloids, has stimulated the study of their pharmacological properties. Triptolide, an important bioactive diterpenoid in *T. wilfordii*, has a variety of pharmacological activities, such as antitumor, anticancer, immunosuppressive, and anti-inflammatory activities [5,6,7,8]. In recent years, the biosynthetic pathway of triptolide has been widely studied. Many biosynthetic enzyme genes have been isolated and functionally characterized, including not only the upstream pathway genes, such as *TwHMGS* [9], *TwDXS1/2* [10,11], *TwDXR* [10,12], *TwHDR* [13], *TwIDI* [14,15], and *TwGGPPS1/4/8* [16,17], but also the downstream pathway genes, such as *TwTPS7/7v2/9/9v2* [18,19], *TwTPS27/27v2* [18,19], and *TwCYP728B70* [20]. However, less is known about the regulatory mechanism of key genes in triptolide biosynthesis in *T. wilfordii*, as compared with the progress of triptolide biosynthetic enzyme genes.

Miltiradiene synthase (MS) genes, *TwTPS27* and *TwTPS27v2* (highly homologous genes), encode miltiradiene synthase to convert *normal*-copalyl diphosphate (*normal*-CPP) to miltiradiene [18,19]. Miltiradiene is an important precursor of triptolide biosynthesis and is also the key intermediate in the biosynthesis of many other medicinally active diterpenoids, such as tanshinones, carnosol, and carnosic acid [21]. Previous studies showed that MS genes were specifically expressed at a high level in roots, in which triptolide levels were enriched [17,18,19,20]. Furthermore, the transcriptional expression of MS genes was upregulated by exogenous application of methyl jasmonate (MeJA), which was consistent with the JA-inducible triptolide biosynthesis [17,19,20]. These results indicate that MS genes play an important role in JA-inducible triptolide biosynthesis in roots. Therefore, given its important role in triptolide biosynthesis, the MS genes were chosen to explore the regulatory mechanism of key genes in triptolide biosynthesis in our study. 

*Cis*-regulatory elements present in gene promoters are essential for understanding the regulatory mechanism of gene expression [22,23]. Jin et al. [24] cloned the *Zmap* promoter from *Zea mays* and characterized several *cis*-regulatory elements present in its promoter, such as the MeJA-responsive element, drought-responsive element, and low-temperature element. Subsequently, β-glucuronidase (GUS) fluorometric assays confirmed the hypothesis that these elements strongly influence the expression of *Zmap* under MeJA, drought, and low-temperature conditions. Yan et al. [25] isolated the promoter of *SaBS* from *Santalum album* and predicted several salicylic acid (SA)-responsive elements existed in its promoter, and the GUS fluorometric assays demonstrated that the activity of the *SaBS* promoter was upregulated significantly by SA treatment. Therefore, the isolation of the *TwTPS27a/b* promoters and functional characterization of their *cis*-regulatory elements will help understand the regulatory mechanism of *TwTPS27a/b* in triptolide biosynthesis.

It is well known that many transcription factors (TFs) regulate the biosynthesis of plant metabolites by binding to the TF binding sites in their key pathway gene promoters. For example, SmbHLH10 TF regulates the diterpenoid tanshinone biosynthesis by binding to the T/G-box and G-box-like motif in the promoters of key enzyme genes, such as *SmDXS2*, *SmCPS1,* and *SmCPS5*, in *Salvia miltiorrhiza* [26]. FhMYB21L2 TF participates in flavonol biosynthesis by directly binding to the MYBPLANT and MYBCORE elements of flavonol biosynthetic gene *FhFLS2* in *Freesia hybrida* [27]. OpWRKY1 TF negatively regulates the camptothecin biosynthesis by binding to the W-box within the promoter of the key gene *OpCPR* in *Ophiorrhiza pumila* [28]. These findings also suggest that the TF binding sites present in key gene promoters play important roles in the regulation of secondary metabolites biosynthesis in plants. Therefore, the TF binding sites present in the promoters of *TwTPS27a/b* need to be investigated.

TGA TFs belong to the basic leucine zipper (bZIP) gene family, play crucial roles in stress response and multiple biological processes [29,30], and regulate plant metabolites biosynthesis by interacting with the TGA-binding sites, either the as-1 element (TGACG-motif) or the as-1-like element (TGACGT sequence) in the promoters of their target genes [30,31,32,33]. In *Artemisia annua*, AaTGA6 is involved in the SA signaling pathway to regulate sesquiterpene lactone artemisinin biosynthesis through interacting with the TGACG-motif within the promoter of artemisinin-regulatory gene *AaERF1* [34]. In *Oryza sativa*, OsTGAP1 regulates JA-inducible diterpenoid phytoalexin biosynthesis through binding to the TGACGT sequence present in the promoters of its target genes, *OsDXS3* and *OsKSL4* [35]. In *Salvia miltiorrhiza*, SmbZIP7 and SmbZIP20, two TGA-type TFs, are predicted to be responsible for regulating diterpenoid tanshinone biosynthesis [36]. At present, only one TF has been reported to regulate the terpenoid metabolism in *T. wilfordii*. Han et al. [37] demonstrated that *TwTGA1* overexpression increased the yields of triptolide in *T. wilfordii* cells and further increased after Methyl jasmonate (MeJA) treatment. However, the regulatory mechanism of TwTGA1 in JA-inducible triptolide biosynthesis has not been elucidated, and its target gene is also unknown.

In this study, the two highly homologous sequences of MS genes, *TwTPS27a* and *TwTPS27b*, were distinguished, and their promoters were isolated and analyzed. In silico analysis of their promoter sequences revealed that many *cis*-regulatory elements were predicted, including hormone-responsive elements and transcription factor (TF) binding sites. To identify the activity of various regulatory regions of the *TwTPS27b* promoter, 5′-deletion assays were performed in transiently transformed tobacco plants under normal and MeJA conditions. In addition, yeast one-hybrid and GUS transactivation assays confirmed the interaction between the *TwTPS27a/b* promoters and the TwTGA1 TF. All these results will help understand the regulatory mechanism of *TwTPS27a/b*, as two target genes of TwTGA1, in JA-inducible triptolide biosynthesis.

## 2. Results

### 2.1. Isolation of Promoters and Prediction of Cis‑Regulatory Elements

To identify the regulatory mechanisms of MS genes, *TwTPS27a* and *TwTPS27b*, in triptolide biosynthesis, the 1496 bp- and 1862 bp-length promoter fragments upstream of the start codon (ATG) were isolated using the methods of SiteFinding-polymerase chain reaction (PCR) and fusion primer and nested integrated-PCR (FPNI-PCR) (Figure S1). Before this, to distinguish the sequences of these two highly-homologous genes (sharing 98.4% nucleotide sequence identity), the full-length coding sequence (CDS), gDNA, and 5′/3′-untranslated region (5′/3′-UTR) sequences of *TwTPS27a* and *TwTPS27b* were also successfully obtained by PCR amplification (Figure S2) and series of sequence alignment (Figures S3–S6). After alignment analysis (Figure S7), it was determined that the 1496 bp-length promoter sequence belongs to the *TwTPS27a* gene, whereas the 1862 bp-length promoter sequence belongs to the *TwTPS27b* gene, respectively. Then, the sequences of *TwTPS27a* and *TwTPS27b,* including promoter, full-length CDS, gDNA, and 5′/3′-UTR sequences, were submitted to the National Center for Biotechnology Information (NCBI) database with accession numbers MN901987 and MN901988, respectively. Moreover, the transcription start sites (TSSs) of *TwTPS27a* and *TwTPS27b* were both identified to be the first base Adenine (A) (both located at −124 bp upstream of the start codon, assigned as position +1) at the end of 5′-UTR (Figure S7). So, relative to TSS, the promoter regions of *TwTPS27a* and *TwTPS27b* were defined as −1372 to +124 bp (1496 bp in length) and −1738 to +124 bp (1862 bp in length), respectively.

**Figure 1:**
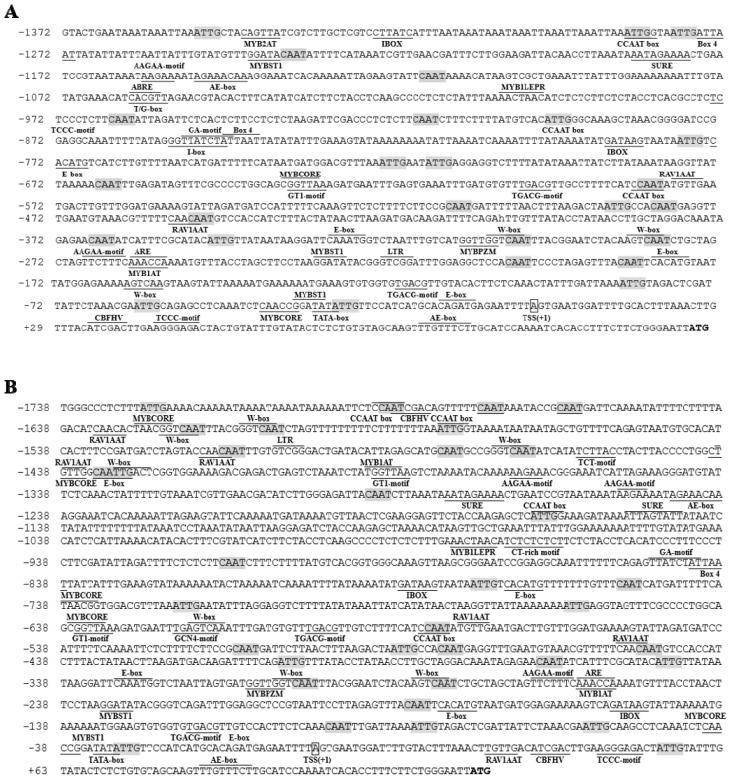
Putative *cis*-regulatory elements present in the *TwTPS27a* promoter (**A**) and the *TwTPS27b* promoter *(***B**) using the PLACE and PlantCARE databases. Numbers indicate the positions relative to the transcription start site (TSS, assigned as position +1, with a box). Putative CAAT-box elements are shown in shaded. Underlined and overlined sequences represent predicted *cis*-regulatory elements.

Based on the New PLACE and PlantCARE databases, putative *cis*-elements involved in the regulation of gene expression were predicted in the *TwTPS27a/b* promoters. The prediction results showed that large numbers of *cis*-regulatory elements were predicted (Figure 1 and Table S1). Some typical core promoter elements were detected, such as TATA-boxes (−30 to −33 bp in both promoters, relative to the TSS) and CAAT-boxes (many positions within both promoters) (Figure 1). Several *cis*-elements known to be involved in hormone response, including TGACG-motif (MeJA-responsiveness), ABRE (Abscisic acid-responsiveness), RAV1AAT (ethylene-responsiveness), PYRIMIDINEBOXHVEPB1 (Gibberellin-responsiveness), and W-box (salicylic acid-responsiveness), were recognized in their promoters (Figure 1 and Table S1). And some TF binding sites were also identified, such as TGACG-motif (binding site of bZIP or TGA TFs), E-box (binding site of bHLH or MYC2 TFs), MYB recognition elements (binding site of MYB TFs), RAV1AAT (binding site of AP2/ERF TFs), and W-box (binding site of WRKY TFs) (Figure 1 and Table S1). Lots of root-specific expression elements and several organ-specific elements were also predicted (Table S1). In addition, several light-responsive elements and stress-related response elements, such as fungal elicitor responsive elements, pathogen- and salt-induced elements, low-temperature response elements, heat shock elements, and dehydration-responsive elements, were also identified in this study (Table S1).

### 2.2. Promoter Deletion Analysis under Normal and MeJA Treatment Conditions

**Figure 2:**
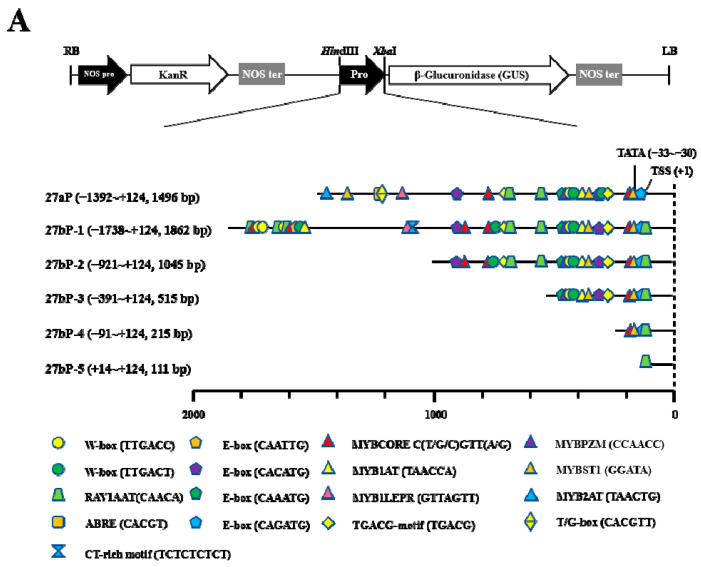

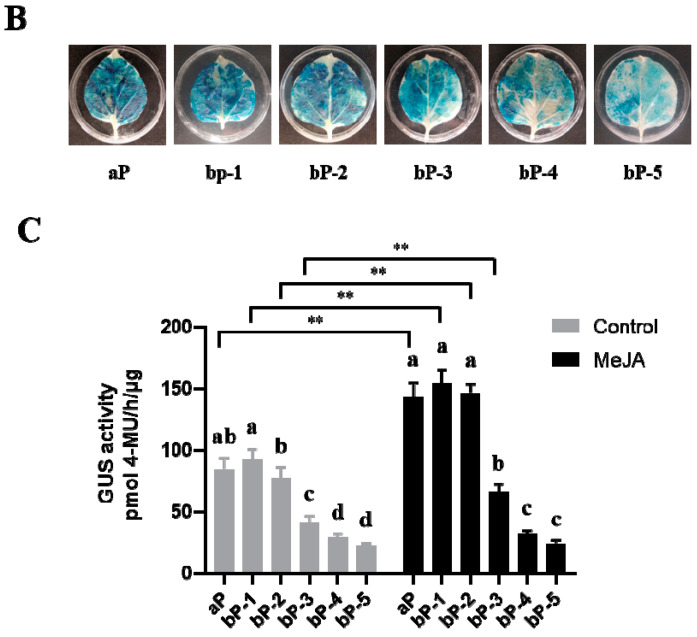
Activity analysis of the full-length promoter of *TwTPS27a/b* and a series of 5′-deletion fragments of the *TwTPS27b* promoter in transiently transformed tobacco plants under normal and MeJA treatment conditions. (**A**) Schematic diagram of the promoter::GUS constructs. Some important *cis*-regulatory elements are shown in this schematic diagram. The transcription start site (TSS) was defined as +1. The full-length sequence of the *TwTPS27a* promoter (27aP, −1392~+124, 1496 bp) and a series of 5′-truncated sequences of the *TwTPS27b* promoter, including 27bP-1 (−1738~+124, 1862 bp), 27bP-2 (−921~+124, 1045 bp), 27bP-3 (−391~+124, 515 bp), 27bP-4 (−91~+124, 215 bp), and 27bP-5 (+14~+124, 111 bp) were fused to the GUS gene to form a series of promoter::GUS constructs. (**B**) GUS staining was observed in agroinfiltrated tobacco leaves by carrying different promoter::GUS constructs. aP, 27aP::GUS; bP-1, 27bP-1::GUS; bP-2, 27bP-2::GUS; bP-3, 27bP-3::GUS; bP-4, 27bP-4::GUS; bP-5, 27bP-5::GUS. (**C**) GUS activity of various promoter deletion fragments in transiently transformed tobacco plants under normal and MeJA treatment conditions. Tobacco leaves infiltrated with *A. tumefaciens* harboring various promoter::GUS constructs were sprayed with 100 μM MeJA or water (as control) 48 h after agroinfiltration. GUS activity was determined 24 h after MeJA or water treatment conditions and was expressed as pmol 4-MU/h/μg protein. The data represent the means ± SD of three independent experiments. Different lower-case letters indicate statistically significant differences at *p* < 0.05 (Duncan’s multiple range tests). Statistical differences between MeJA treated and normal (control) conditions were determined by Student’s *t*-test (**, *p* < 0.01).

Comparing the two promoter sequences of MS genes *TwTPS27a/b*, we found that the −1115 to +124 bp (1239 bp) region of *TwTPS27a* promoter and −1088 to +124 bp (1212 bp) region of *TwTPS27b* promoter were highly homologous (93.0% identity, Figure S8), indicating that many common *cis*-regulatory elements are present in both promoters (Figure 2A). Therefore, to determine the activities of various regulatory regions of the two MS gene promoters, only the *TwTPS27b* promoter harboring more *cis*-regulatory elements was selected for 5′-deletion analysis. A series of promoter fragments, including 27aP, 27bP-1, 27bP-2, 27bP-3, 27bP-4, and 27bP-5, were then obtained by PCR and cloned into the plasmid pBI121 harboring the GUS gene to construct various promoter::GUS vectors (Figure 2A and Figure S9). In 5′-deletion assays, all of these promoter::GUS constructs were separately transformed into the tobacco leaves to test promoter activity using the *Agrobacterium*-mediated transient expression system. First, GUS staining showed that the agroinfiltrated tobacco leaves with various promoter::GUS constructs all produced a blue color, implying that all tested promoter fragments were functional promoter sequences and could drive the expression of the GUS reporter gene (Figure 2B). Subsequently, the GUS activities of these promoter deletion fragments in transiently transformed tobacco plants were determined under normal and MeJA treatment conditions.

Under normal conditions, the full-length promoter of *TwTPS27b* (27bP-1, −1738/+124) showed the strongest GUS activity, whereas that of the full-length promoter of *TwTPS27a* (27aP, −1392/+124) was slightly lower than 27bP-1, but not significant (Figure 2C). Moreover, the promoter activities progressively decreased from 27bP-1 to 27bP-5 in transiently transformed tobacco leaves (Figure 2C). Notably, when 27bP-1 was deleted from −921 bp (27bP-2, −921/+124) to −391 bp (27bP-3, −391/+124), it resulted in a loss of quantitative GUS activity (−39.14% of 27bP-1 activity, Figure 2C). The results suggested that the 531 bp sequence (−921 to −391 bp) might be the core region of the *TwTPS27b* promoter. Under MeJA stress, the promoter activities of 27aP, 27bP-1, 27bP-2, and 27bP-3 significantly improved by 1.69-, 1.68-, 1.89-, and 1.59-fold, respectively, compared with their corresponding controls (Figure 2C), whereas that of 27bP-4 and 27bP-5 showed no significant change. These results indicate that one or more MeJA-responsive elements exist in the *TwTPS27a/b* promoters, not in the −391 to +124 bp region (27bP-4) of *TwTPS27b* promoter. In addition, when the promoter sequence of 27bP-1 was deleted from −921 (27bP-2) to −391 bp (27bP-3) and from −391 (27bP-3) to −91 bp (27bP-4), both caused a loss of quantitative GUS activity, −52.06% and −21.91% of 27bP-1 activity, respectively (Figure 2C). These results imply that the MeJA-responsive elements might exist in the −921 to −391 bp and −391 to −91 bp regions of the *TwTPS27b* promoter.

### 2.3. TwTGA1 Specifically Binds to the TGACG-motif in the TwTPS27a/b Promoters

Recently, TwTGA1, a MeJA-inducible TF, was reported to modulate triptolide biosynthesis in *T. wilfordii* cells [37]. However, the direct target gene of TwTGA1 has not been identified in triptolide biosynthesis. It is well known that TGA TFs recognize the TGACG-motif (TGA-binding site) within the promoters of their target genes [30,31,32]. In silico analysis of the *TwTPS27a/b* promoters revealed that two TGACG-motifs were found in the *TwTPS27b* promoter, one located in the potential core region of −921 to −391 bp and the other located in the region of −391 to −91 bp (Figure 1A and Figure 2A). That was similar to the *TwTPS27a* promoter (Figure 1B and Figure 2A). So, both promoters have two TGA-binding sites (TGACG-motifs), indicating that the *TwTPS27a/b* promoters have the potential to be bound by TwTGA1.

**Figure 3:**
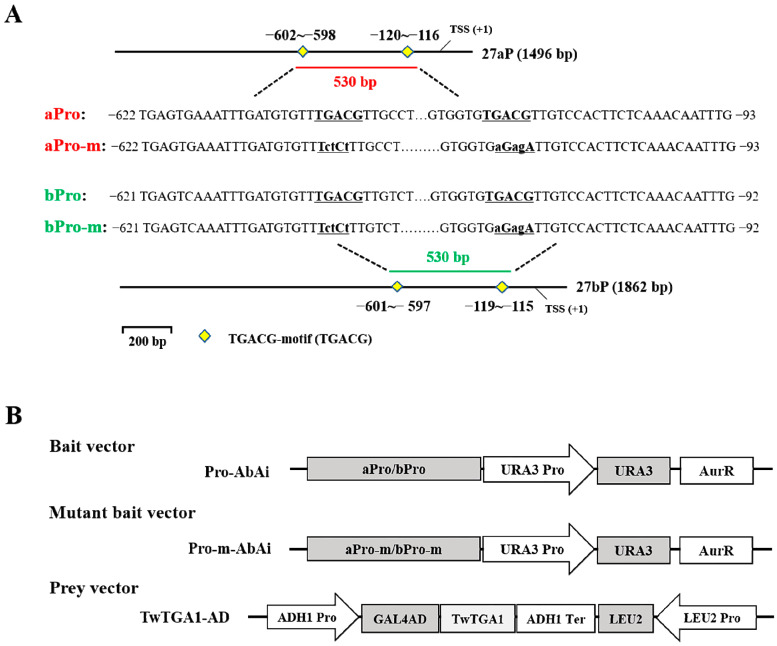

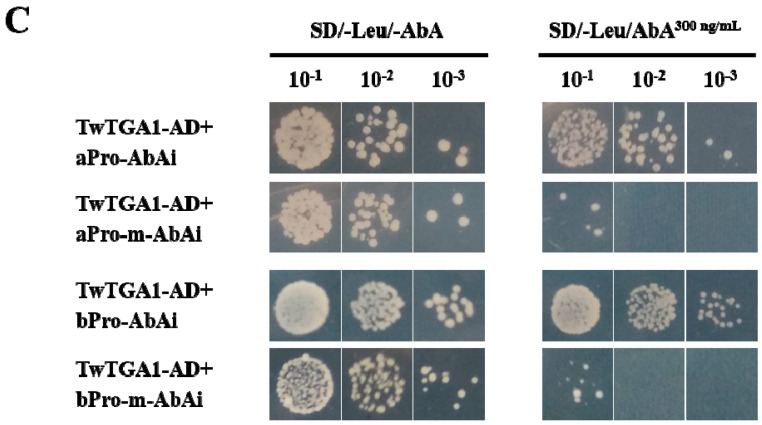
Yeast one-hybrid (Y1H) assays showing that TwTGA1 specifically binds to the TGACG-motif present in the *TwTPS27a/b* promoters. (**A**) Schematic diagrams of the promoter and mutant promoter fragments in the *TwTPS27a* (−622~−93, 530 bp) and *TwTPS27b* (−621~−92, 530 bp) used for the construction of the bait and mutant bait vectors. The yellow diamond represents the TGACG-motif. aPro and bPro are both 530 bp containing two ‘TGACG’ elements, while aPro-m and bPro-m are mutated forms of aPro and bPro (‘TGACG’ mutated to ‘TctCt’ by PCR cloning). (**B**) Schematic diagrams of the bait, mutant bait, and prey vectors used for Y1H assays. (**C**) Y1H assays showing the interaction between TwTGA and the TGACG-motif present in the *TwTPS27a/b* promoters, based on the ability of the transformed yeast strains to grow on SD/−Leu/AbA^300ng/mL^ medium with gradient dilution (1/10, 1/100, 1/1000). The transformants grown on SD/−Leu/−AbA plate were used as positive controls for transformants growth. Positive transformants were confirmed by spotting yeast cells onto agar medium of SD/−Leu with 300 ng/mL AbA. These assays were repeated three times with similar results.

To verify the binding of TwTGA1 to the TGACG-motif present in the *TwTPS27a/b* promoters, yeast one-hybrid assays were performed. The aPro and bPro promoter fragments (530 bp truncated fragments of the *TwTPS27a/b* promoters harboring two ‘TGACG’ sequences, Figure 3A) and the aPro-m and bPro-m mutant promoter fragments (where two ‘TGACG’ sequences were both mutated to ‘TctCt’, Figure 3A) were all inserted into the pAbAi vector, which contained the *Aureobasidin A* resistance (AurR) reporter gene, to generate the bait vectors (aPro-AbAi and bPro-AbA, Figure 3B) and mutated bait vectors (aPro-m-AbAi and bPro-m-AbA, used as the negative controls, Figure 3B). The full-length CDS of TwTGA1 was fused to the yeast GAL4 activation domain (GAL4 AD) of the pGADT7-Rec2 vector to generate the prey vector, TwTGA1-AD (Figure 3B). After introducing the prey vector TwTGA1-AD into the yeast strains harboring the mutant bait vectors, aPro-m-AbAi or bPro-m-AbAi, it was found that the growth of the transformed yeast strains was inhibited on an SD/−Leu plate under 300 ng/mL concentration of aureobasidin A (AbA), which is a potent and unique yeast antibiotic (Figure 3C). But when transformed with the prey vector TwTGA1-AD, the yeast bait strains harboring aPro-AbAi or bPro-AbAi vectors were still able to grow normally on an SD/−Leu/AbA^300^ plate (Figure 3C). These findings suggested that TwTGA1 was indeed able to bind the TGACG-motif in the *TwTPS27a/b* promoters, which was consistent with the expectation of TGA TFs.

### 2.4. TwTGA1 Can Activate the TwTPS27a/b Promoters

**Figure 4:**
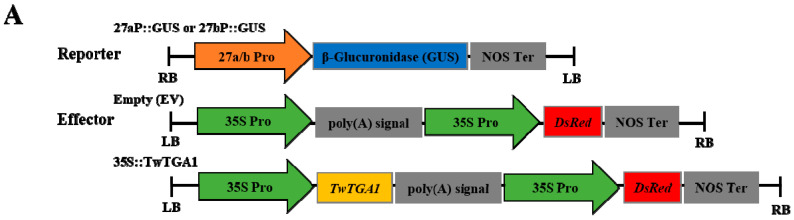

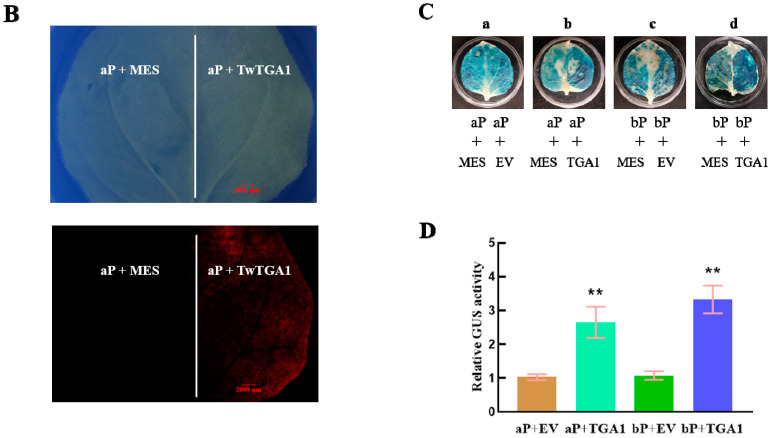
Activation of the *TwTPS7a/b* promoters by transient overexpression of TwTGA1 in tobacco leaves. (**A**) Schematic diagrams of the reporter and effector vectors for GUS staining and GUS fluorimetric assays. LB and RB, left and right T-DNA borders. (**B**) Red fluorescent signals in transiently transformed tobacco leaf. Top panel: bright field; bottom panel: RFP fluorescence. Scale bar: 2000 μm. aP, 27aP::GUS; TwTGA1, 35S::TwTGA1; MES, 2-(N-morpholino)ethanesulfonic acid (MES) buffer. (**C**) The GUS staining analysis of the infiltrated tobacco leaves. Different combinations of the mixed bacterial suspension were infiltrated into the left and right sides of the midrib in the same tobacco leaves, respectively. a, bacterial suspension harboring 27aP::GUS+EV was infiltrated into the right side of the midrib, while 27aP::GUS+MES into the left side of the midrib (as the control) in the same tobacco leaf. b, bacterial suspension harboring 27aP::GUS+35S::TwTGA1 was infiltrated into the right side of the midrib, while 27aP::GUS+MES into the left side of the midrib (as the control) in the same tobacco leaf. c, bacterial suspension harboring 27bP::GUS+EV was infiltrated into the right side of the midrib, while 27bP::GUS+MES into the left side of the midrib (as the control) in the same tobacco leaf. d, bacterial suspension harboring 27bP::GUS+35S::TwTGA1 was infiltrated into the right side of the midrib, while 27bP::GUS+MES into the left side of the midrib (as the control) in the same tobacco leaf. aP, 27aP::GUS; bP, 27bP::GUS; TwTGA1, 35S::TwTGA1; EV, empty vector; MES, MES buffer. (**D**) GUS activity analysis showing that TwTGA1 activates the promoters of *TwTPS27a/b*. The value of relative GUS activity was measured by the ratio of GUS activity in the right side of the midrib to that of the left side of the midrib in the same tobacco leaf. All data presented here are means±SD of three biological replicates, and these assays were repeated at least three times with similar results. Statistical significance was determined using Student’s *t*-test (**, *p* < 0.01).

To examine whether TwTGA1 directly activates the promoters of *TwTPS27a/b*, GUS staining and GUS transactivation assays were performed in transiently transformed tobacco leaves. The schematic diagrams of the reporters and effectors used for GUS transactivation assays are shown in Figure 4A. A DsRed (*Discosoma* sp. red fluorescent protein) gene present in the effectors was used for identifying whether 35S::TwTGA1 or empty vector (EV) could be transiently overexpressed in tobacco leaves (Figure 4B). The GUS activity analysis showed that higher GUS staining intensity was observed in the infiltrated tobacco leaves co-transformed with 35S::TwTGA1 (effector) and 27aP::GUS/27bP::GUS (reporters), than their corresponding controls, which was only transformed to 27aP::GUS or 27bP::GUS (Figure 4C, b and d). Their GUS activities also significantly increased to 2.65- and 3.33-fold, respectively (Figure 4D). The empty vector (EV) had no interaction with the reporters either 27aP::GUS or 27bP::GUS (Figure 4C, a and c; Figure 4D). Therefore, these results revealed that TwTGA1 positively regulated the activities of the *TwTPS27a/b* promoters.

## 3. Discussion

To explore the molecular basis for the regulation of MS genes, *TwTPS27a* and *TwTPS27b*, in triptolide biosynthesis, the promoters of *TwTPS27a* (1496 bp, −1392 to +124 bp) and *TwTPS27b* (1862 bp, −1738 to +124 bp) were isolated from *T. wilfordii* using the methods of SiteFinding-PCR and FPNI-PCR (Figure S1). In silico analysis of the *TwTPS27a* and *TwTPS27b* promoters revealed that many common *cis*-regulatory elements existed in their promoter regions (the sequences between *TwTPS27a* promoter from −1115 to +124 bp and *TwTPS27b* promoter from −1088 to +124 bp were highly homologous, with 93.0% identity, Figure S8) (Figure 2A), suggesting that both promoters have similar regulation traits. In the present study, the promoter activity of 27aP (full-length promoter of *TwTPS27a*, 1496 bp) was not significantly different from that of 27bP-1 (full-length promoter of *TwTPS27b*, 1862 bp) in driving the GUS expression in transiently transformed tobacco leaves (Figure 2C). Furthermore, the promoter activities of 27aP and 27bP-1 were both significantly increased by MeJA treatment, to a similar fold (1.69- and 1.68-fold, respectively), compared to their corresponding controls (Figure 2C). This result also implied that *TwTPS27a* and *TwTPS27b* are MeJA-responsive genes, which is consistent with recent studies that the application of MeJA could increase the transcript level of MS genes in *T. wilfordii* suspension cells [19,20].

Previous studies have shown that exogenous application of MeJA upregulated the transcription levels of triptolide biosynthetic pathway genes, and the accumulation of triptolide in *T. wilfordii* was also significantly increased by MeJA treatment [17,19,20]. In silico analysis of the *TwTPS27a/b* promoters led to the identification of two MeJA-responsive elements, TGACG-motifs (Figure 1 and Figure 2A and Table S1), which have been reported to have a role in MeJA response [38,39]. Xiong et al. [40] isolated the promoter of key gene *PtAOS1* from *Poncirus trifoliata* and demonstrated that a TGACG-motif present in the core region −532 to +1 bp of *PtAOS1* promoter was probably responsible for MeJA response. In this study, the 5′-deletion assays in transiently transformed tobacco plants under normal and methyl jasmonate (MeJA) conditions showed that the deletion of −921 to −391 bp in the *TwTPS27b* promoter caused a loss of quantitative GUS activity (−39.14% of 27bP-1 activity, Figure 2C) and the MeJA-mediated stress induction of GUS activity was also significantly decreased (−52.06% of 27bP-1 activity, Figure 2C). These results implied that the sequence of −921 to −391 bp might be the core region of *TwTPS27b* promoter and the TGACG-motif, which was found in this potential core region (−921 to −391 bp), might be responsible for the *TwTPS27b* promoter in the response of MeJA stress.

The analysis of *cis*-regulatory elements present in gene promoters is helpful for understanding the molecular mechanism of *TwTPS27a/b* genes expression in triptolide biosynthesis. In this study, several fungal elicitor-responsive elements (TTGACC) were predicted in the *TwTPS27a/b* promoters (Table S1). Our previous studies showed that the fungal elicitors *Glomerella cingulata* and *Collectotrichum gloeosporioides* could increase the content of triptolide by 2.24 and 1.93-fold, respectively, compared to the control [41]. A number of root-specific expression elements were also identified, including ROOTMOTIFTAPOX1 (ATATT), OSE1ROOTNODULE (AAAGAT), and OSE2ROOTNODULE (CTCTT) (Table S1), implying that *TwTPS27a/b* might be the root-specific expression genes. Previous studies showed that the highest expression levels of *TwTPS27* are in the root, whereas very low in the stem, young-, and mature leaf. This might be responsible for the enrichment of triptolide in the root of *T. wilfordii* [17,18,19,20]. Moreover, this apparent discrepancy in their expression patterns is of great significance to the discovery of the candidate biosynthetic pathway genes of plant secondary metabolites. Hansen et al. [18] specifically mined the transcriptome of the root to discover candidate terpene synthase (TPS) genes involved in the biosynthesis of diterpenoids in *T. wilfordii*, complementing the candidate genes from the earlier reported leaf-specific transcriptome library [42] and leading to the identification of the first two enzyme genes (*TwTPS9* and *TwTPS27*) downstream of triptolide biosynthesis. In addition, apart from the MeJA-responsive element (TGACG-motif), some other elements are associated with hormones were also found in the *TwTPS27a/b* promoters (Table S1), such as ABRE, RAV1AAT, W-box, and PYRIMIDINEBOXHVEPB1/PYRIMIDINEBOXOSRAMY1A elements, participating in the signal response of abscisic acid, ethylene, salicylic acid, and gibberellin, respectively. This implied that *TwTPS27a/b* might be involved in various phytohormone signaling pathways. However, whether these hormones can affect the expression of *TwTPS27a/b*, thereby regulating the accumulation of triptolide in *T. wilfordii,* is still unclear, and it is worthy of further study.

**Figure 5:**
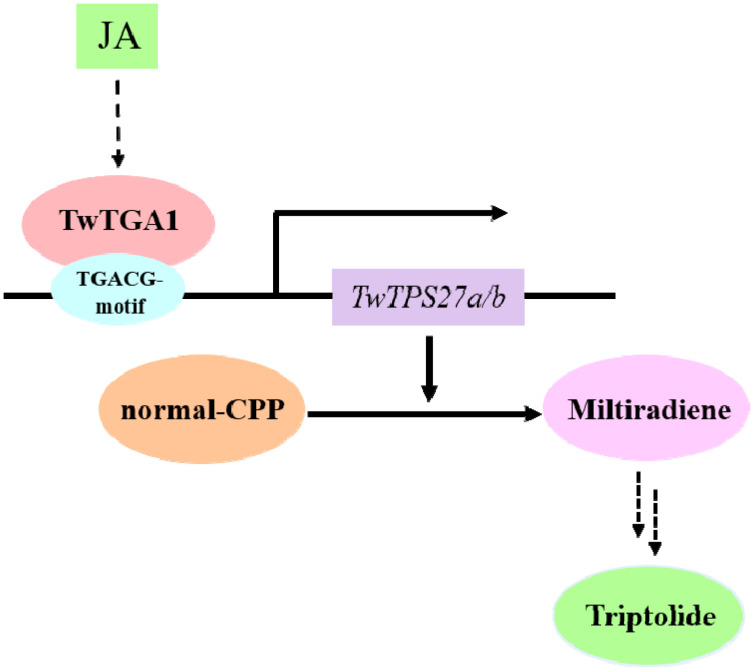
The proposed model of TwTGA1 TF in regulating the methyl jasmonate (MeJA)-mediated triptolide biosynthesis through the activation of *TwTPS27a/b*.

The TGACG-motif found in the potential core region of *TwTPS27b* promoter was not only the MeJA-responsive element but also the TF binding site, in which it is recognized by TGA TFs involved in the regulation of secondary metabolism in plants, such as AaTGA6 [34] and OsTGAP1 [35]. In *T. wilfordii*, TwTGA1, a MeJA-responsive TF, was reported to enhance the yields of triptolide in *T. wilfordii* cells [37]. However, although the EMSA assays showed that the TwTGA1 protein could specifically bind to the TGACG-motif and TGACGT sequence within the promoters of *MPO1* and *PMT*, two key enzyme genes of pyridine alkaloids biosynthesis in tobacco BY-2 cells [37], the molecular mechanism and the target gene of TwTGA1 in the regulation of triptolide biosynthesis were still not elucidated. In this study, Yeast one-hybrid (Y1H) assays further verified the specific binding of TwTGA1 to the TGACG-motif present in the promoters of *TwTPS27a*/*b* in yeast cells (Figure 3C). In addition, the GUS transactivation assays further demonstrated the activation of the *TwTPS27a*/*b* promoters by TwTGA1 in transiently transformed tobacco leaves (Figure 4C,D). These results confirmed the interaction between the TwTGA1 TF and the *TwTPS27a/b* promoters, indicating that the MS genes, *TwTPS27a* and *TwTPS27b*, are two target genes of TwTGA1. Therefore, based on previous reports and these findings here, we deduced that TwTGA1 regulates the MeJA-inducible triptolide biosynthesis through the activation of *TwTPS27a/b* in *T. wilfordii*, thereby increasing triptolide content, and proposed a working model (Figure 5). In brief, MeJA induces the expression of *TwTGA1*. TwTGA1 TF directly binds to the promoters of *TwTPS27a/b* harboring two TGACG-motifs and activates its expression, converting *normal*-copalyl diphosphate to miltiradiene, thereby promoting the accumulation of triptolide in *T. wilfordii*.

In addition, multiple putative TF binding sites also exist in the promoters of *TwTPS27a/b*, including E-box, MYB recognition element (MRE), W-box, and RAV1AAT elements (Figure 1 and Table S1). Among them, the E-box elements (CANNTG), also known as MYC motifs, could be bound by the bHLH [43,44,45] and MYC2 TFs (members of bHLH subgroup IIIe) [46,47]. The MREs were demonstrated to be bound by MYB TFs [48,49]. The W-box elements, TTGAC(C/T), are the specific *cis*-elements binding with WRKY TFs [50,51]. The RAV1AAT (CAACA) motif could be recognized by AP2/ERF TFs [52,53]. The existence of multiple putative TF binding sites in the promoters of *TwTPS27a*/*b* suggests that the regulation mechanism of *TwTPS27a/b* in triptolide biosynthesis is a relatively complex process. However, whether these important TF binding sites present in the *TwTPS27a/b* promoters could be recognized by other TFs that may regulate the biosynthesis of triptolide in *T. wilfordii*, such as the bHLH, MYC2, MYB, WRKY, and AP2/ERF TF family members, still needs further study.

In *T. wilfordii*, much of the observed pharmacological activity can be attributed to the presence of triptolide, which was the first of many diterpenoids identified, attracting interest due to the spectrum of bioactivities [18]. However, the low content of triptolide in *T. wilfordii* limits its wide application in medicine. This study provides an important theoretical basis for screening and verifying the candidate TFs, which could increase the content of triptolide in transgenetic *T. wilfordii* cells or hairy roots in the future by metabolic engineering.

## 4. Materials and Methods

### 4.1. Plant Materials, Growth Conditions

Hairy root cultures of *T. wilfordii* were previously induced from root explants, as described in previous work [54]. Tobacco (*Nicotiana benthamiana*) plants for agroinfiltration were grown under 16 h day/8 h night (24 ± 1 °C) conditions in a plant growth chamber for six weeks.

### 4.2. Isolation of the TwTPS27a/b Genes

The total RNA and genomic DNA were extracted from the hairy root of *T. wilfordii* according to the instruction manuals of the Plant Total RNA Extraction Kit (DNA-free) and Plant Genomic DNA Extraction Kit (Biospin, Shanghai, China). The first-strand cDNA synthesis was performed according to the manufacturer’s protocol of PrimeScript™ II 1st Strand cDNA Synthesis Kit (TaKaRa, Dalian, China). Local BLAST (Basic Local Alignment Search Tool) was conducted with the query sequence of *TwTPS27* (KU948698), and two candidate sequences were obtained from two transcriptome libraries of *T. wilfordii*, SRX472292 and SRX202900, with the highest score (bits) (Table S2). After sequence alignment, the sense and antisense primers, *TwTPS27*-UTR-F and *TwTPS27*-UTR-R, were designed (Figure S10) in the 5′-untranslated region (5′-UTR) and 3′-untranslated region (3′-UTR) of the candidate genes to amplify the full-length coding sequence (CDS) and gDNA of these two target genes by polymerase chain reaction (PCR). The amplified PCR products were cloned into the pMD19-T vector (TaKaRa, Dalian, China) by the TA cloning procedure. After sequencing and sequence alignment, two different sequences were distinguished and named *TwTPS27a* and *TwTPS27b*. In addition, the 5′/3′-UTR sequences of *TwTPS27a* and *TwTPS27b* were isolated by 5′/3′-rapid amplification of cDNA ends (5′/3′-RACE) according to the protocol of SMARTer RACE 5′/3′ Kit (Clontech, CA, USA). The primers for 1st round PCR (*TwTPS27*-5′-R1-624 and *TwTPS27*-3′-F1-974) and 2^nd^ round PCR (*TwTPS27*-5′-R2-302 and *TwTPS27*-3′-F2-1422) were designed according to the cDNA sequences of *TwTPS27a/b*. The 2nd round PCR products of 5′/3′-RACE were then introduced into the pMD19-T clone vector by TA cloning procedure and sequenced. After sequence alignment, 5′/3′-UTR sequences of *TwTPS27a* and *TwTPS27b* were determined. All primers used here are listed in Table S3.

### 4.3. Cloning and Analysis of the TwTPS27a/b Promoter Sequences

The promoter sequences of *TwTPS27a* and *TwTPS27b* were isolated by the methods of SiteFinding-PCR [55] and fusion primer and nested integrated-PCR (FPNI-PCR) [56]. In this study, we conducted two SiteFinding-PCR assays. In the first experiment, three primers in nested positions, 27pro-1, 27pro-2, and 27pro-3, were designed according to the gDNA sequences of *TwTPS27a/b* and used to isolate the 5′-flanking sequences of *TwTPS27a/b*. In the second experiment, based on a short 5′-flanking sequence cloned in the first experiment, three new primers in nested positions, 27pro-4, 27pro-5, and 27pro-6, were designed to clone a longer/new 5′-flanking sequence (Figure S1A). Then the specific sense primers, Pro-1-F and Pro-R, for cloning the full-length promoter sequence were designed based on the longer 5′-flanking sequence cloned in the second experiment of SiteFinding-PCR and the 5′UTR sequence of *TwTPS27a/b*, leading to isolating a 1496 bp-length sequence upstream of the start codon (Figure S1C). After that, to isolate a new 5′-flanking sequence, which differs from the 1496 bp sequence, another three primers in nested positions, XXR494, XXR410, and XXR358, were designed according to the 5′-flanking sequence cloned in the second experiment of SiteFinding-PCR and applied to another method called FPNI-PCR, thereby cloning a longer and new 5′-flanking sequence (Figure S1B). Then we finally obtained a longer and different 5′-flanking sequence, 1862 bp-length sequence upstream of the start codon (Figure S1C), with the specific primers, Pro-2-F and Pro-R. Finally, the 5′-flanking sequences of *TwTPS27a/b* were identified through sequences alignments with the 5′-UTR and cDNA sequences of the *TwTPS27a/b* genes. The PCR reaction systems and procedures of these two methods are shown in Table S4–S7. All primers used here are shown in Table S3.

The putative *cis*-regulatory elements in the promoter sequences of *TwTPS27a/b* were predicted using the New PLACE [57] (https://www.dna.affrc.go.jp/PLACE/?action=newplace (accessed on 22 September 2020)) and PlantCARE [58] (http://bioinformatics.psb.ugent.be/webtools/plantcare/html/ (accessed on 22 September 2020)) databases. The transcription start sites (TSSs) of *TwTPS27a/b* were determined by comparing the 5′ flanking sequences with the 5′-UTR sequences of *TwTPS27a/b*.

### 4.4. Construction of a Series of Promoters::GUS Vectors

The full-length promoter fragments of *TwTPS27a/b* and a series of 5′-truncated fragments of *TwTPS27b* promoter were PCR-amplified using the different forward primers (27aP-F, 27bP-1-F, 27bP-2-F, 27bP-3-F, 27bP-4-F, and 27bP-5-F) and a single reverse primer 27P-R (listed in Table S3). The amplified fragments were cloned into the binary vector pBI121, replacing the cauliflower mosaic virus 35S (CaMV35S) promoter using NovoRec^®^ plus One step PCR Cloning Kit (Novoprotein, Shanghai, China). These corresponding promoter::GUS vectors were named aP, bp-1, bP-2, bP-3, bP-4, and bP-5. After sequencing and verification by double restriction-enzyme digestion and PCR, all of these constructs were transformed into *Agrobacterium tumefaciens* GV3101 (pSoup-p19) (Weidi, Shanghai, China) for further transient expression. 

### 4.5. Agrobacterium-Mediated Transient Expression in Tobacco Leaves and MeJA Treatment

*Agrobacterium*-mediated transient expression assays were performed according to a previous study [59] with minor modification. The harvested *Agrobacterium* harboring a series of promoter::GUS constructs were resuspended in MES buffer (10 mM MES, pH 5.6, 10 mM MgCl_2_, 2% (w/v) sucrose and 120 μM acetosyringone), adjusted to an OD_600_ of 0.6 and incubated at room temperature for 3 h before infiltration in tobacco leaves. Each bacterial suspension was infiltrated into the intact leaves of six-week-old tobacco plants using a needleless syringe. After agroinfiltration, tobacco plants were first grown in darkness for 12 h and then maintained in a growth chamber under a 16/8 h day/night cycle at 25 °C for 60 h. Then, the infiltrated leaves were harvested for GUS activity analysis.

For MeJA treatment, tobacco plant leaves infiltrated with *A. tumefaciens* harboring a series of promoter::GUS constructs were sprayed with 100 μM MeJA or water as control at 48 h after agroinfiltration. The infiltrated leaves were then frozen in liquid nitrogen at 24 h after MeJA treatment and stored at −70 °C for GUS fluorometric assay. All experiments were repeated three times.

### 4.6. GUS Staining and GUS Fluorimetric Assays

GUS staining assays were performed as described by Li et al. [60], with modification. The treated tobacco leaves were incubated in GUS staining solution (50 mM sodium phosphate buffer (50 mM NaH_2_PO4·2H_2_O and 50 mM Na_2_HPO_4_·12H_2_O, pH 7.0), 10 mM Na_2_EDTA·2H_2_O (pH 8.0), 0.1% Triton X-100, 0.5 mM K_3_[Fe(CN)_6_], 0.5 mM K_4_[Fe(CN)_6_]·3H_2_O, and 1 mg/mL X-Gluc (5-bromo-4-chloro-3-indolyl-β-D-glucuronic acid)) for 16 h at 37 °C in the dark, and then were decolored with 70% (*v*/*v*) ethanol at 37 °C before being photographed.

Fluorimetric assays for GUS activity were carried out according to the method previously described with modifications [61]. The transient tobacco leaf (50 mg) was homogenized in 500 µL GUS extraction buffer containing 10 mM Na_2_EDTA·2H_2_0 (pH 8.0), 50 mM sodium phosphate buffer (pH 7.0), 0.1 % Triton X-100, 0.1 % (w/v) sodium dodecyl sulfate, and 0.1 % β-mercaptoethanol. After centrifugation at 12,000 rpm (4 °C) for 10 min. The supernatant (10 μL) was diluted with 90 µL of extraction buffer and mixed with 100 μL pre-warmed (37 °C) Gus reaction solution (2 mM 4-methyl-umbelliferyl-β-D-glucuronide in extraction buffer) and incubated at 37 °C for 1 h. The reaction was terminated by adding 1 mL stop buffer (0.2 M Na_2_CO3). The fluorescence level of 4-methylumbelliferone (4-MU) was determined using an F-4500 fluorescence spectrophotometer (Hitachi, Tokyo, Japan) at 365 (excitation) and 455 (emission) nm. The concentration of 4-MU in the range of 31.25 nM to 40 µM was used to generate a standard curve. The total concentration of protein extracts from the transient tobacco leaves was assessed by the Bradford method [62] with bovine serum albumin as standard. GUS activity was measured as pmol of 4-MU generated per hour per μg protein. GUS measurements were repeated at least three times independently with similar results.

### 4.7. The Binding of TwTGA1 to the TGACG-motif Present in the TwTPS27a/b Promoters

To detect whether TwTGA1 binds to the TGACG-motif present in the *TwTPS27a/b* promoters in yeast, the Yeast one-hybrid (Y1H) assays were performed according to the manufacturer’s instruction (Clontech, CA, USA). Two promoter fragments of *TwTPS27a* (−622~−93, 530 bp) and *TwTPS27b* (−621~−92, 530 bp), harboring two TGACG-motifs, were obtained by PCR and named 27aPro and 27bPro, respectively. They were then ligated into pAbAi (Clontech, CA, USA) to generate the bait vectors (27aPro-AbAi and 27bPro-AbAi). Moreover, two ‘TGACG’ sequences present in the 27aPro and 27bPro were both mutated to ‘TctCt’ by PCR and also ligated into pAbAi, thereby generating the mutant bait vectors (aPro-m-AbAi and bPro-m-AbA). The coding sequence of *TwTGA1* (GenBank Accession number: MN080495) was amplified by PCR and fused to the yeast GAL4 activation domain (GAL4 AD) of pGADT7-Rec2 (Clontech, CA, USA), thereby generating the prey vector TwTGA1-AD. The bait vectors were then linearized and integrated into the genome of Y1H Gold yeast stain, selected on SD/-Ura agar medium plate, which was cultured at 30 °C for 3 days. Next, the prey vector TwTGA1-AD was introduced into the yeast cells previously transformed with the bait/mutant bait vectors, including aPro-AbAi, aPro-m-AbAi, bPro-AbAi, and bPro-m-AbAi. The concentration of positively transformed yeast cells that harbor different combinations of the bait and prey vectors were adjusted to OD600 ≈ 1.0 and serially diluted 1/10, 1/100, 1/1000 in sterile ddH_2_O. The serial dilution of transformed yeast cells was cultured on SD/−Leu medium plates with an optimal concentration of aureobasidin A (AbA) to examine any protein-DNA interactions. The plates were incubated at 30 °C for 3 days. All primers used are listed in Table S3.

### 4.8. Activation of the TwTPS7a/b Promoters by TwTGA1 in Tobacco Leaves

To verify whether TwTGA1 could activate the *TwTPS27a/b* promoters, GUS transactivation assays were performed on tobacco leaves. The ORF of TwTGA1 was cloned by PCR (primers see Table S3) and introduced into a modified vector pCAMBIA1302 containing an RFP reporter gene, thereby generating the effector 35S::TwTGA1. The corresponding empty vector (EV) was used as a negative control. The promoter expression vectors, 27aP::GUS and 27bP::GUS, as the reporters. All of these constructs were separately transformed into *A. tumefaciens* GV3101 (pSoup-p19). The bacterial cultures harboring the effectors (35S::TwTGA1 or EV) and reporters (27aP::GUS or 27bP::GUS) were resuspended in MES buffer to OD_600_ of 1.2 and 0.6, respectively, and mixed in the ratio of 1:1 (v:v) before infiltration. Each mixed bacterial suspension was infiltrated into the left and right sides of the midrib in the same leaf, respectively. The GUS fluorimetric activities of these infiltrated tobacco leaves were measured until 72 h after infiltration. The value of relative GUS activity was measured by the ratio of GUS activity in the right side of the midrib to that of the left side of the midrib in the same tobacco leaf. Three independent experiments were performed.

### 4.9. Statistical Analysis

All data were presented as means±SD of three biological replicates. Statistical analyses were carried out using the SPSS23.0 (SPSS, IBM, USA) software running one-way ANOVA according to Duncan’s Multiple Range Test (DMRT), taking *p* < 0.05 as statistically significant. Statistical differences between MeJA treated and normal (control) conditions were determined by Student’s *t*-test (*, *p* < 0.05; **, *p* < 0.01).

## 5. Conclusions

Miltiradiene synthase genes, *TwTPS27a* and *TwTPS27b*, are the key genes in triptolide biosynthesis. To identify the regulatory mechanisms of *TwTPS27a* and *TwTPS27b*, the 1496 bp- and 1862 bp-length promoter sequences of these two highly homologous genes were isolated and characterized. Both promoters have many similar regulatory elements and displayed the same responses to MeJA treatment. The −921 to −391 bp sequence harboring a TGACG-motif, MeJA-responsive element, is the potential core region of the *TwTPS27b* promoter. Yeast one-hybrid and GUS transactivation assays confirmed that MS genes *TwTPS27a/b* are the targets of TwTGA1, a MeJA-inducible TGA TF upregulating triptolide biosynthesis in *T. wilfordii*. Our findings provide important information for elucidating the regulatory mechanism of MS genes or other triptolide biosynthetic pathway genes in JA-inducible triptolide biosynthesis.

## Data Availability

The data that support the findings of this study are available from the corresponding author, [X.Z.], upon reasonable request.

## Supplementary Materials

The following are available online at https://www.mdpi.com/2223-7747/10/2/418/s1, Table S1: Prediction of the *cis*-regulatory elements in the *TwTPS27a/b* promoters, Table S2: The results of local BLAST for query sequence of *TwTPS27* (KU948698), Table S3: Primers used in this study, Table S4: Reaction system of SiteFinding-PCR, Table S5: Reaction procedures of SiteFinding-PCR, Table S6: Reaction system of fusion primer and nested integrated-PCR (FPNI-PCR), Table S7: Reaction procedures of FPNI-PCR, Figure S1: Cloning of the 5′-flanking sequences of *TwTPS27a* and *TwTPS27b*, Figure S2: Identification of the PCR products for the full-length coding sequence (CDS), gDNA and 5′/3′-RACE of *TwTPS27a* and *TwTPS27b*, Figure S3: The full-length CDSs and protein sequences alignment between *TwTPS27a* and *TwTPS27b*, Figure S4: Sequence alignment between the full-length CDS and gDNA of *TwTPS27a*, Figure S5: Sequence alignment between the full-length CDS and gDNA of *TwTPS27b*, Figure S6: Sequence alignment between 5′/3′-RACE sequences and CDSs of *TwTPS27a/b*, Figure S7: Sequence alignment for the determination of the *TwTPS27a* and *TwTPS27b* promoters. Figure S8: Sequence alignment between *TwTPS27a* promoter from −1115 to +124 bp and *TwTPS27b* promoter from −1088 to +124 bp, Figure S9: Verification of various promoter::GUS vectors, Figure S10: Primers designed for cloning the CDSs and gDNA sequences of *TwTPS27a/b*.
