# Supplementary material for: Isolation and Functional Characterization of the Promoters of Miltiradiene Synthase Genes, TwTPS27a and TwTPS27b, and Interaction Analysis with the Transcription Factor TwTGA1 from Tripterygium wilfordii
Source: Plants (Basel). 2021 Feb 23;10(2):418. doi: 10.3390/plants10020418 (PMC7926782; doi:10.3390/plants10020418)
Supplement: Supplementary file 1 [file plants-10-00418-s001.pdf]

Supplementary Materials

Table S1. Prediction of the *cis*-regulatory elements in the *TwTPS27a/b* promoters

Site name	Numbers of		Sequence	Function
	27aP	27bP		
TGACG-motif	2	2	TGACG	Cis-acting regulatory element involved in the meja-responsiveness
PYRIMIDINEBOXHVEPB1	1	1	TTTTTTCC	Required for GA induction, pyrimidine box
PYRIMIDINEBOXOSRAMY1A	1	0	CCTTTT	Gibberellin-response cis-element, pyrimidine box
W-box	3	7	TTGAC(T/C)	WRKY recognition site, salicylic acid (SA)-responsiveness
E-box	3	4	CANNTG	MYC recognition sequence, meja-responsiveness
T/G-box	1	0	CACGTT	MYC recognition sequence, meja-responsiveness
MYB1AT	1	2	(T/A)AACCA	MYB recognition site, dehydration-responsive
MYB2AT	1	0	TAACTG	MYB recognition sequence, dehydration-responsive
MYB1LEPR	1	1	GTTAGTT	Cis elements of Myb1
MYBPZM	1	1	CCAACC	Core of consensus maize P (myb homolog) binding site
MYBST1	3	2	GGATA	Core motif of mybst1 (a potato MYB homolog) binding site
MYBCORE	2	5	C(G/C)GTT(A/G)	CNGTTR, MYB recognition site, dehydration-responsive
ABRE	1	0	ACGTG	Abscisic acid responsiveness
RAV1AAT	2	6	CAACA	The AP2-like domain recognize the CAACA motif; ethylene-responsive
CAATBOX1	27	35	CAAT	Play a key role in transcription initiation
CTRMCAMV35S	0	1	TCTCTCTCT	CT-rich motif, can enhance gene expression
AAGAA-motif	1	1	GAAAGAA	Light responsive element
AE-box	2	2	AGAAACAA	Part of a module for light response
Box 4	2	1	ATTAAT	Part of a conserved DNA module involved in light responsiveness
GA-motif	1	1	ATAGATAA	Part of a light responsive element
GT1-motif	1	2	GGTTAA	Light responsive element
I-box	1	0	TAGATAACC	Part of a light responsive element
GATABOX	11	16	GATA	Light regulated, and tissue specific expression
IBOX	2	2	GATAAG	Light responsive element
IBOXCORE	5	6	GATAA	Light responsive element
SORLIP1AT	1	1	GCCAC	One of "Sequences Over-Represented in Light-Induced Promoters (sorlips) in Arabidopsis
SORLIP2AT	0	2	GGGCC	One of "Sequences Over-Represented in Light-Induced Promoters (sorlips)

SORLIP5AT	1	0	GAGTGAG	Light responsive element
TBOXATGAPB	0	1	ACTTTG	Mutations in the "Tbox" resulted in reductions of light-activated gene transcription
TCCC-motif	2	1	TCTCCCT	Part of a light responsive element
TCT-motif	0	1	TCTTAC	Part of a light responsive element
ACGTATERD1	6	4	ACGT	Required for etiolation-induced expression
ARE	1	1	TGGTTT	Essential for the anaerobic induction
CBFHV	1	2	RYCGAC	Dehydration-responsive element (DRE) binding proteins
CCAATBOX1	3	4	CCAAT	HSE (Heat shock element); CCAAT box
CURECORECR	3	2	GTAC	Copper-response element
ELRECOREPCRP1	1	4	TTGACC	Fungal elicitor responsive element
GT1GMSCAM4	6	9	GAAAAA	Plays a role in pathogen- and salt-induced
LTRECOREATCOR15	1	1	CCGAC	Low-temperature response
SURE1STPAT21	1	2	AATAGAAAA	Sucrose responsive element (sure)
SURECOREATSULTR11	1	2	GAGAC	Core of sulfur-responsive element (SURE) found in the promoter of SULTR1
AACACOREOSGLUB1	1	1	AACAAAC	endosperm-specific expression
AACACOREOSGLUB1	1	1	AACAAAC	endosperm-specific expression
GCN4_motif	0	1	TGAGTCA	cis-regulatory element involved in endosperm expression
OSE1ROOTNODULE	3	4	AAAGAT	organ-specific elements, activated in infected cells of root nodules
OSE2ROOTNODULE	5	8	CTCTT	organ-specific elements, activated in infected cells of root nodules
ROOTMOTIFTAPOX1	14	12	ATATT	root-specific expression
RYREPEATBNNAPA	1	2	CATGCA	Seed-specific expression

Table S2. The results of local BLAST for query sequence of *TwTPS27* (KU948698)

Transcriptome	Matched sequence	Score(bits)	E-value	Length(bp)
SRX472292	*comp34029_c1_seq3*	1095	0.0	2562
SRX202900	*Tw_comp37092_c0_seq1_len=2086*	1180	0.0	2086
SRX472292	*comp27356_c0_seq1*	295	5e-080	375
SRX202900	*Tw_comp37092_c0_seq1_len=2086*	256	9e-068	2086

Table S3. Primers used in this study

Primers	Sequence (5' to 3')	Use for
Full-length cDNA and gDNA PCR		
TwTPS27-UTR-F	AAGTTTGTTTCTTGCATCCAAAATC	cDNA-PCR and
TwTPS27-UTR-R	GATGTCCAAACTACTTATTTGCTAA	gDNA-PCR
5'/3'-RACE PCR		
TwTPS27-5'-R1-624	CTCTTTGGCTTCATCTAAAATAACT	1st 5'-RACE PCR
TwTPS27-5'-R2-302	TCAGAGGTGGACGCAAGCAATC	2nd 5'-RACE PCR
TwTPS27-3'-F1-974	CCAAAGCCAGAATAGCAGCAAC	1st 3'-RACE PCR
TwTPS27-3'-F2-1422	GCTACTCTACTGGTCGTCCGTCAT	2nd 3'-RACE PCR
Site Finding-PCR		
Site Finder-1	CACGACACGCTACTCAACACACCACCTCGCACAGCGTCCTCAACCTGCAGGIBIHIGCTC	Initial PCR
Site Finder-2	CACGACACGCTACTCAACACACCACCTCGCACAGCGTCCTCAAGCGGCCGCNNNNNNGCTC	Initial PCR
Site Finder-3	CACGACACGCTACTCAACACACCACCTCGCACAGCGTCCTCAACTCGAGNNNNNNAGCT	Initial PCR
SFP1	CACGACACGCTACTCAACAC	1st PCR
SFP2	ACTCAACACACCACCTCGCACAGC	2nd PCR
SFP3	CACCTCGCACAGCGTCCTCA	3rd PCR
27pro1	CGTAGGATGCGGAACCTCAATGC	1st PCR
27pro2	GCAATCTCTTGGCATCTTGCCTC	2nd PCR
27pro3	TGTAGGGAGTGTTCAAGGAGTCG	3rd PCR
27pro4	ATCATTGCGGAAGAAAAGAGAACTT	1st PCR
27pro5	TCATCCAAACAAGTCATTCAACATA	2nd PCR
27pro6	GCTGCCAGGGGCGAAACT	3rd PCR
FPNI-PCR		
FP1	GTAATACGACTCACTATAGGGCACGCGTGGTNTCGASTWTSGWGTT	1st PCR
FP2	GTAATACGACTCACTATAGGGCACGCGTGGTNGTCGASWGANAWGAA	1st PCR
FP3	GTAATACGACTCACTATAGGGCACGCGTGGTWGTGNAGWANCANAGA	1st PCR
FP4	GTAATACGACTCACTATAGGGCACGCGTGGTAGWGNAGWANCAWAGG	1st PCR
FP5	GTAATACGACTCACTATAGGGCACGCGTGGTNGTAWAASGTNTSCAA	1st PCR
FP6	GTAATACGACTCACTATAGGGCACGCGTGGTNGACGASWGANAWGAC	1st PCR
FP7	GTAATACGACTCACTATAGGGCACGCGTGGTNGACGASWGANAWGAA	1st PCR

FP8	GTAATACGACTCACTATAGGGCACGCGTGGTGTNCGASWCANAWGTT	1st PCR
FP9	GTAATACGACTCACTATAGGGCACGCGTGGTNCAGCTWSCTNTSCTT	1st PCR
FSP1	GTAATACGACTCACTATAGGGC	2nd PCR
FSP2	ACTATAGGGCACGCGTGGT	3rd PCR
XXR494	CCCGTTTAGCTTTGCCCAATGTGAC	1st PCR
XXR410	TGAAGAGGGAGAGAGGCGTGAGGTA	2nd PCR
XXR358	AGAGGGGCTTGAGGTAGAAGATGAT	3rd PCR
Full-length Promoter cloning		
Pro-1-F	GTACTGAATAAATAAATTAAATTGCTACAGTTATCG	sense primer
Pro-2-F	TGGGCCCTCTTTATTGAAAACAAAAAT	sense primer
Pro-R	AATTCCCAGAAGAAAGGTGTGATTTT	antisense primer
Promoter::GUS construction		
27aP-F	GACCATGATTACGCCAAGCTTGTACTGAATAAATAAATTAAATTGCTACAGTTATCG	sense primer
27bP-1-F	GACCATGATTACGCCAAGCTTTGGGCCCTCTTTATTGAAAACAAAAAT	sense primer
27bP-2-F	GACCATGATTACGCCAAGCTTCTCTCTTCAATCTTTCTTTTATGTCAC	sense primer
27bP-3-F	GACCATGATTACGCCAAGCTTTAACCTTGCTAGGACAAATAGAGAAC	sense primer
27bP-4-F	GACCATGATTACGCCAAGCTTATTAAAATTGTAGACTCGATTATTC	sense primer
27bP-5-F	GACCATGATTACGCCAAGCTTTGTACTTTAAACTTGTTGACATCGA	sense primer
27P-R	ACCACCCGGGGATCCTCTAGAAATTCCCAGAAGAAAGGTGTGATTTT	antisense primer
Y1H assays		
aPro-AbAi-F	AAATGATGAATTGAAAAGCTTTGAGTGAAATTTGATGTGTT**TGACG**	bait vector
aPro-AbAi-R	AGCACATGCCTCGAGGTCGACCAAATAGTTTGAGAAGTGTACAA**CGTCA**	bait vector
aPro-m-AbAi-F	AAATGATGAATTGAAAAGCTTTGAGTGAAATTTGATGTGTT**TctCt**	bait vector
aPro-m-AbAi-R	AGCACATGCCTCGAGGTCGACCAAATAGTTTGAGAAGTGTACAA**aGagA**	bait vector
bPro-AbAi-F	AAATGATGAATTGAAAAGCTTTGAGTCAAATTTGATGTGTT**TGACG**	bait vector
bPro-AbAi-R	AGCACATGCCTCGAGGTCGACCAAATTGTTTGAGAAGTGGACAA**CGTCA**	bait vector
bPro-m-AbAi-F	AAATGATGAATTGAAAAGCTTTGAGTCAAATTTGATGTGTT**TctCt**	bait vector
bPro-m-AbAi-R	AGCACATGCCTCGAGGTCGACCAAATTGTTTGAGAAGTGGACAA**aGagA**	bait vector
TwTGA1-AD-F	GCCATGGAGGCCAGTGAATTCATGAACTCTCCGTCCACCC	prey vector
TwTGA1-AD-R	ACGATTCATCTGCAGCTCGAGCTATTCTCCTTTAAACGACTGTGCAC	prey vector
GUS transactivation assays		
TwTGA1-OE-F	TATAAAAAAAAAAAAGAATTCATGAACTCTCCGTCCACCC	effector vector

TwTGA1-OE-R	ATTATTATGGAGAAAAAGCTTCTATTCTCCTTTAAACGACTGTGCAC	effector vector
27aP-F	in section of Promoter::GUS construction	reporter vector
27aP-R	in section of Promoter::GUS construction	reporter vector
27bP-F	in section of Promoter::GUS construction	reporter vector
27bP-R	in section of Promoter::GUS construction	reporter vector

Table S4. Reaction system of Site Finding-PCR

Initial PCR		1st PCR	
Components	Volume	Components	Volume
Genomic DNA	1.0 μL	Initial PCR product	20.0 μL
—	—	GSP1 (10 μM)	1.0 μl
Site Finer(n) (10 μM)	1.0 μl	SF1 (100 μM)	0.4 μl
Premix PrimeSTAR HS	10.0 μl	Premix PrimeSTAR HS	2.5 μl
ddH_2O	8.0 μL	ddH_2O	1.1 μL
Total	20.0 μl	Total	25.0 μl
2nd PCR		3rd PCR	
Components	Volume	Components	Volume
1st PCR product	1.0 μl	Dilution of 2nd PCR product	1.0 μl
GSP2 (10 μM)	1.0 μl	GSP3 (10 μM)	1.0 μl
SF2 (10 μM)	1.0 μl	SF3 (10 μM)	1.0 μl
Premix PrimeSTAR HS	12.5 μl	Premix PrimeSTAR HS	12.5 μl
ddH_2O	9.5 μL	ddH_2O	9.5 μL
Total	25.0 μl	Total	25.0 μl

Table S5. Reaction procedures of SiteFinding-PCR

Reactions	Circles	Conditions of temperature
Initial PCR	1	94°C 5 min
	1	98°C 1 min; 25°C 1 min; 0.3°C /s heat up to 68°C
	1	68°C 10 min
1st PCR	1	94°C 5 min
	30	98°C 10 s; 55°C 5 s; 68°C 4 min
	1	72°C 5 min
2nd PCR	1	94°C 5 min
	30	98°C 10 s; 55°C 5 s; 68°C 3 min
	1	72°C 5 min
3rd PCR	1	94°C 5 min
	30	98°C 10 s; 55°C 5 s; 68°C 2 min
	1	72°C 5 min

Table S6. Reaction system of fusion primer and nested integrated-PCR (FPNI-PCR)

1st PCR		2nd PCR		3rd PCR	
Components	Volume	Components	Volume	Components	Volume
Genomic DNA	1.5 μL	1st PCR product	1.0 μl	Dilution of 2nd PCR product	1.0 μl
GSP1 (10 μM)	0.4 μl	GSP2 (10 μM)	1.0 μl	GSP3 (10 μM)	1.0 μl
FPn (10 μM)	2.0 μl	FSP1 (10 μM)	1.0 μl	FSP2 (10 μM)	1.0 μl
Premix PrimeSTAR HS	12.5 μl	Premix PrimeSTAR HS	12.5 μl	Premix PrimeSTAR HS	12.5 μl
ddH_2O	8.6 μL	ddH_2O	9.5 μL	ddH_2O	9.5 μL
Total	25.0 μl	Total	25.0 μl	Total	25.0 μl

Table S7. Reaction procedures of FPNI-PCR

Reactions	Circles	Conditions temperature
1st PCR	1	95°C 90 s
	2	94°C 10 s, 62°C 30 s, 72°C 2 min
	1	94°C 10 s, 25°C 2 min, 0.2°C/s, 72°C 2 min
	6	94°C 10 s, 62°C 30 s, 72°C 2 min, 94°C 10 s, 62°C 30 s, 72°C 2 min, 94°C 10 s, 44°C 30 s, 72°C 2 min
	1	72°C 5 min
2nd PCR	1	95°C 90 s
	30	94°C 10 s, 62°C 30 s, 72°C 2 min
	1	72°C 5 min
3rd PCR	1	95°C 90 s
	12	94°C 10 s, 62°C 30 s, 72°C 2 min
	1	72°C 5 min

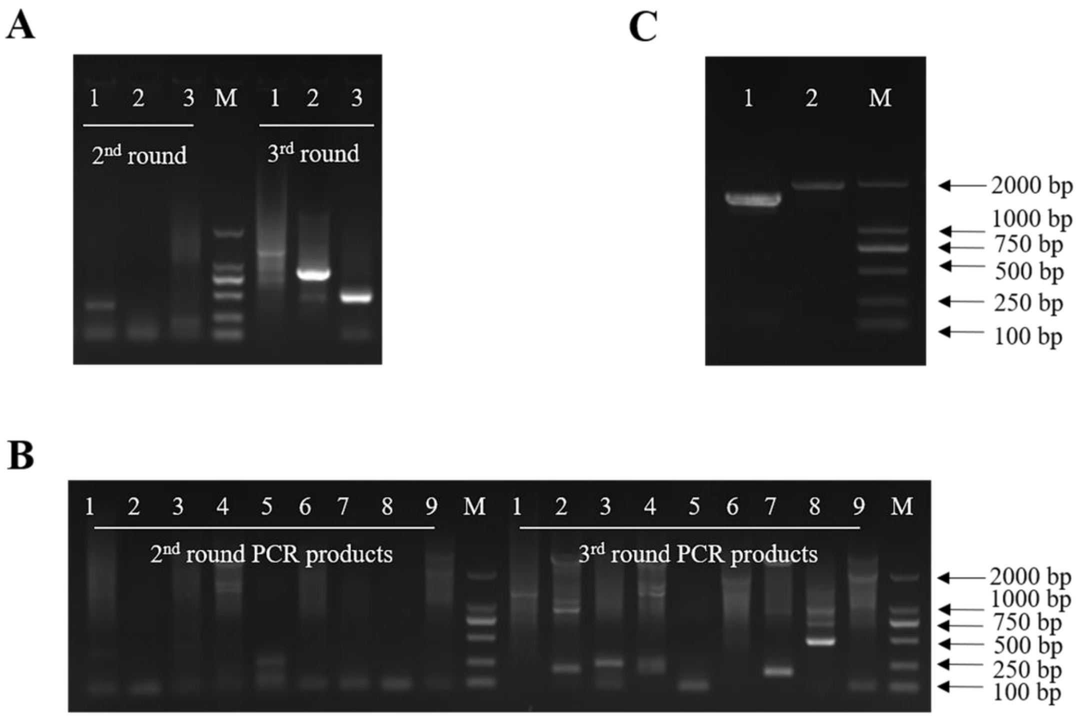

Figure S1. Cloning of the 5'-flanking sequences of *TwTPS27a* and *TwTPS27b*. (A) Identification of the 2nd round and 3rd round PCR products in the SiteFinding-PCR assays. The PCR reaction systems and procedures are shown in Table S4-S5. All primers used here are shown in Table S3. M: DL2000 Marker. (B) Identification of the 2nd round and 3rd round PCR products in the FPNI-PCR assays. The PCR reaction systems and procedures are shown in Table S6-S7. All primers used here are shown in Table S3. M: DL2000 Marker. (C) Identification of the PCR products of 5'-flanking sequence of target genes. 1, the PCR product of the 1496 bp-length 5'-flanking sequence using the specific primers of Pro-1-F and Pro-1-R. 2, the PCR product of the 1862 bp-length 5'-flanking sequence using the specific primers of Pro-2-F and Pro-2-R. All primers used here are shown in Table S3. M: DL2000 Marker.

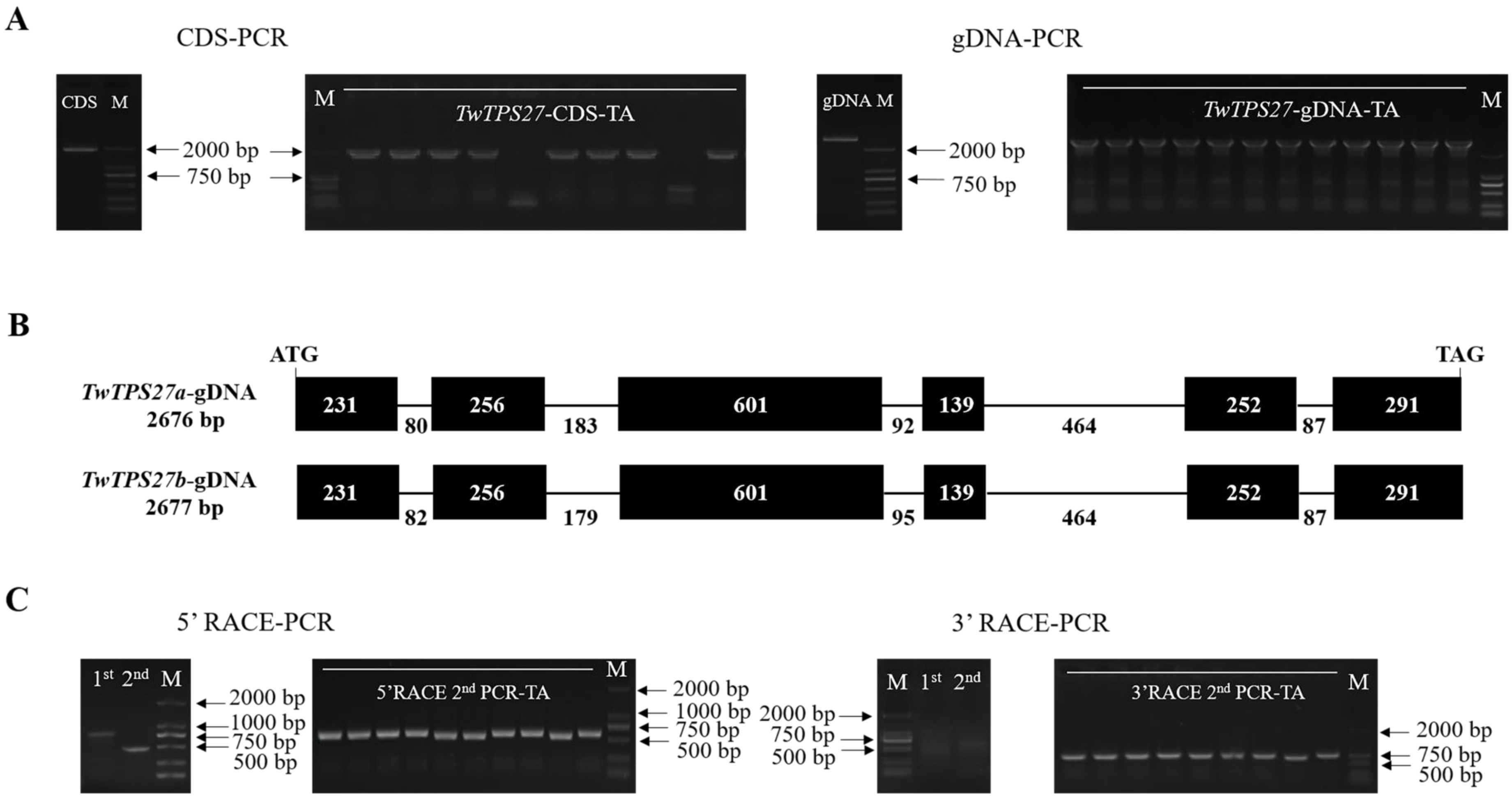

Figure S2. Identification of the PCR products for the full-length coding sequence (CDS), gDNA and 5'/3'-RACE of *TwTPS27a* and *TwTPS27b*. (A) Identification of the PCR product for full-length cDNA and gDNA of *TwTPS27*. Several positive clones of TwTPS27-cDNA-TA and TwTPS27-gDNA-TA were identified. M: DL2000 Marker. (B) Exon-intron structures of *TwTPS27a* and *TwTPS27b*. Exons and introns are represented by black boxes and straight lines, respectively. (C) Electrophoretogram analysis of 5'/3' RACE-PCR. 1st represents 1st-round 5'/3'RACE-PCR products, 2nd represents 2nd-round 5'/3'RACE-PCR products. M: DL2000 Marker. Several positive TA clones of 2nd-round PCR products (5'RACE 2nd PCR-TA and 3'RACE 2nd PCR-TA) for 5'/3'RACE were identified.

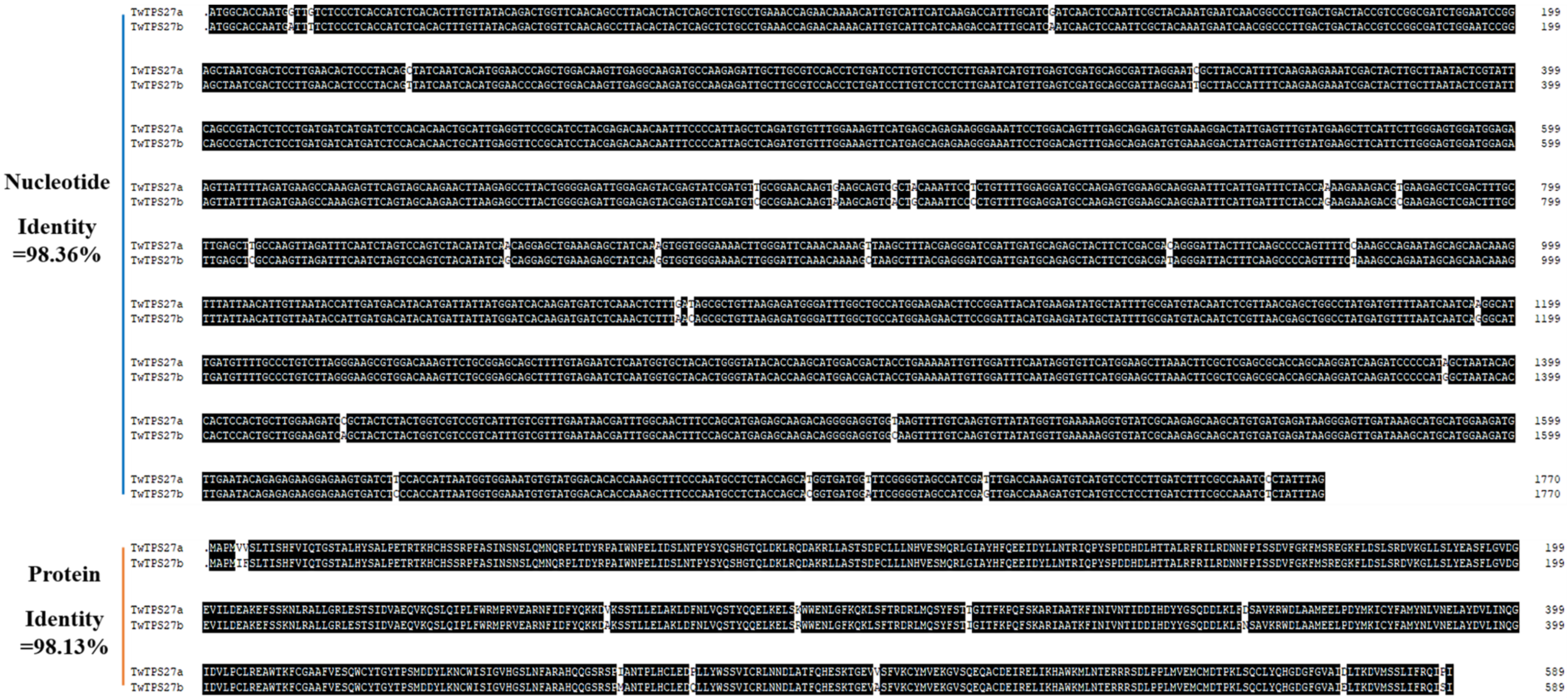

Figure S3. The full-length CDSs and protein sequences alignment between *TwTPS27a* and *TwTPS27b*.

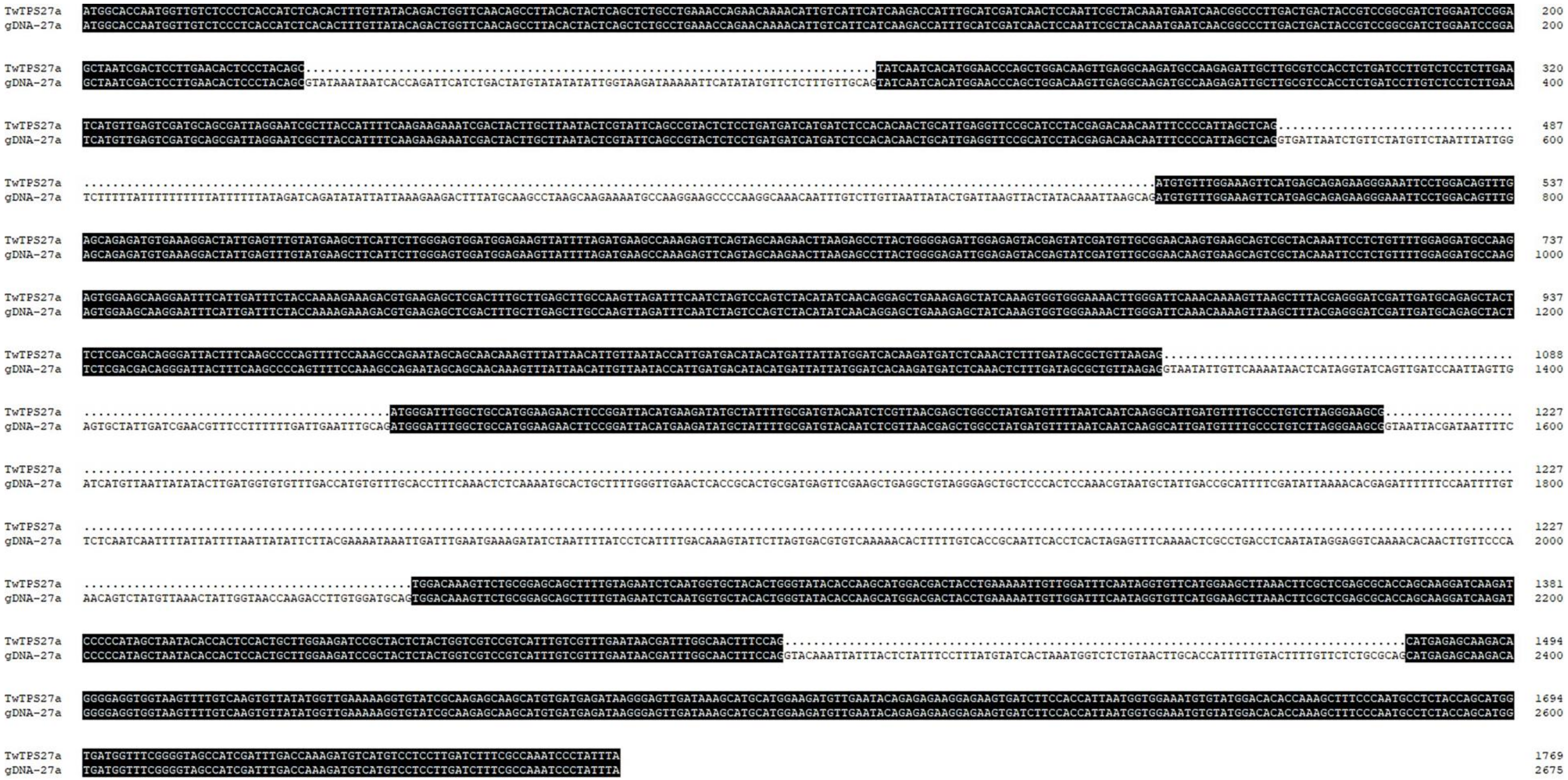

Figure S4. Sequence alignment between the full-length CDS and gDNA of *TwTPS27a*.

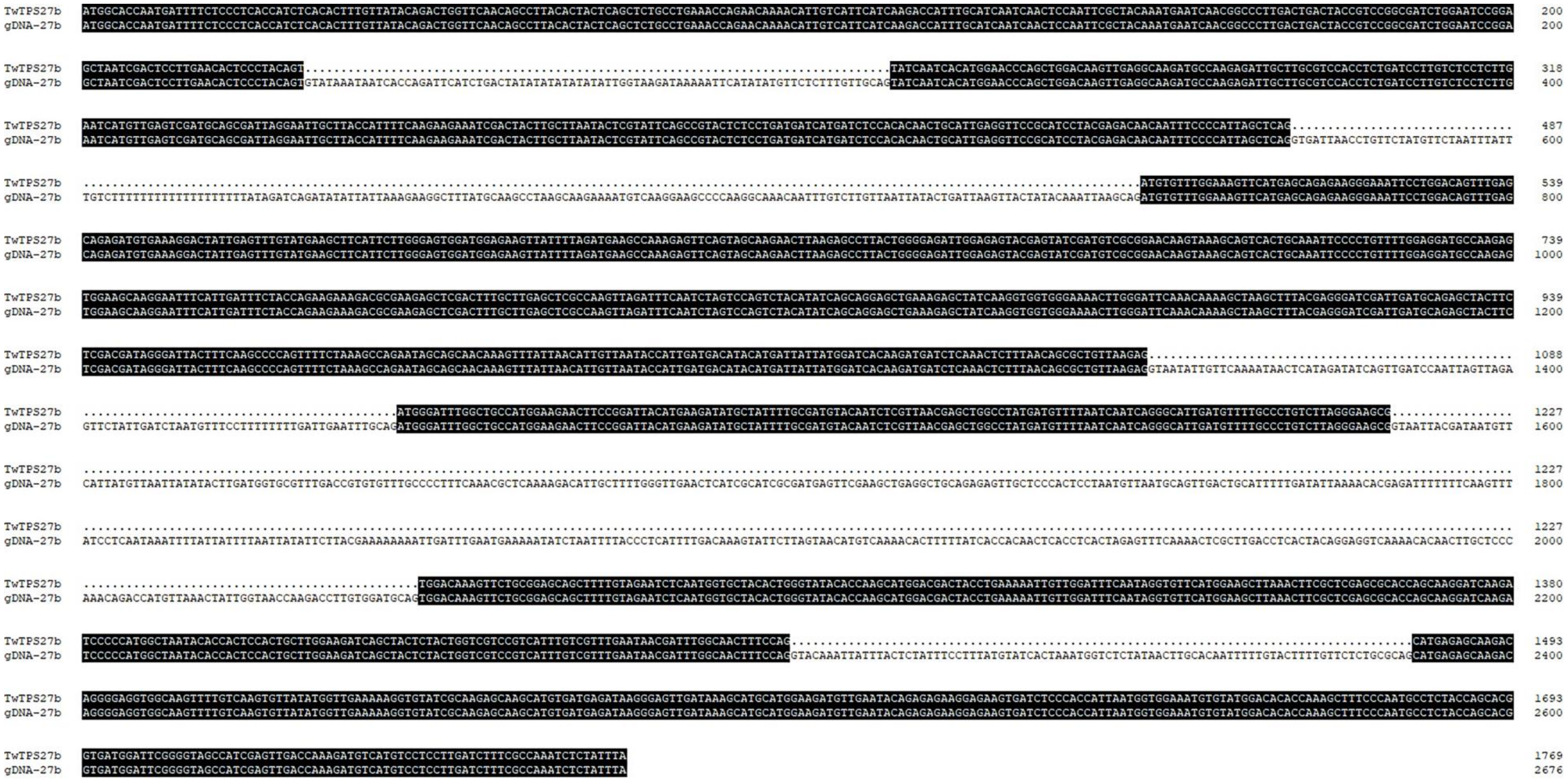

Figure S5. Sequence alignment between the CDS and gDNA of *TwTPS27b*.

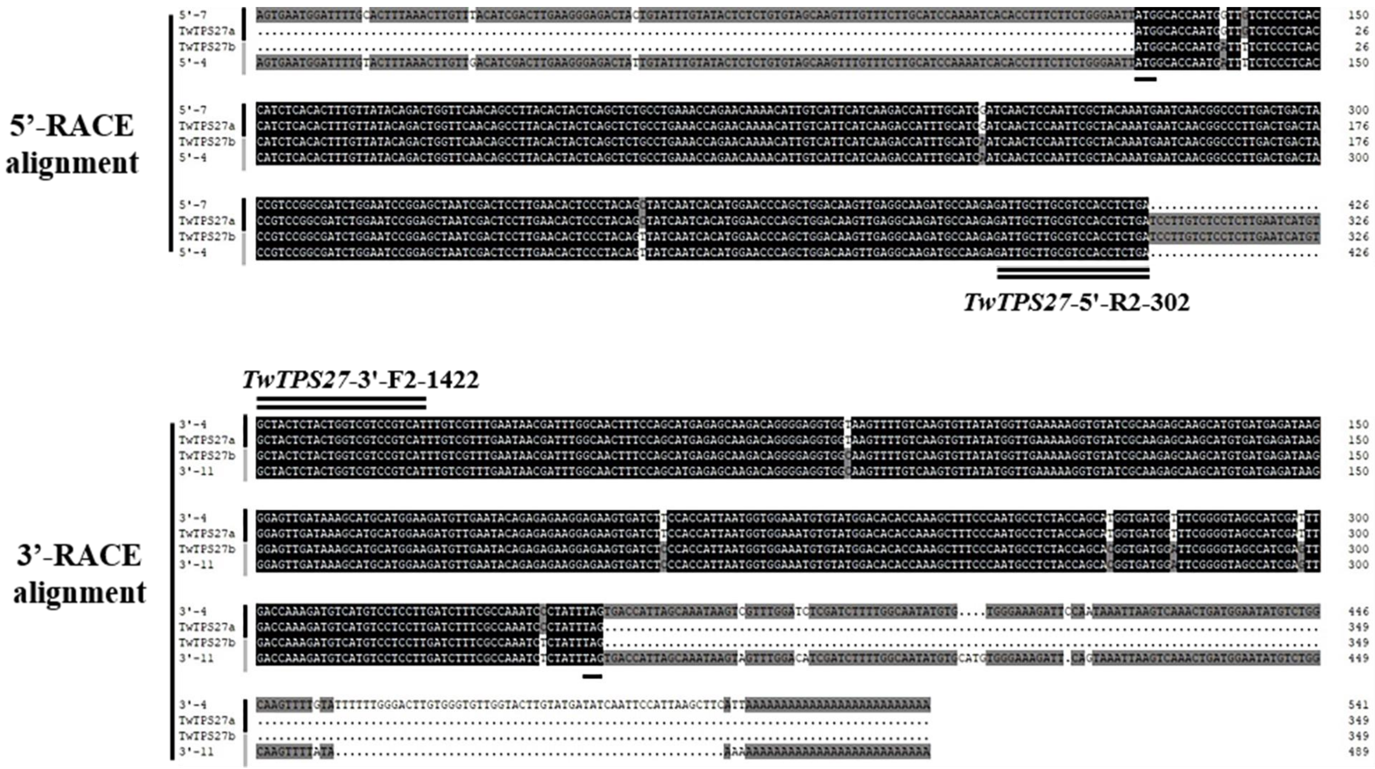

Figure S6. Sequence alignment between 5'/3'-RACE sequences and CDSs of *TwTPS27a/b*. The primers used for 2nd round 5'/3'-RACE PCR are marked with two straight lines. The start codon (ATG) and stop codon (TAG) are marked with a sigle line.

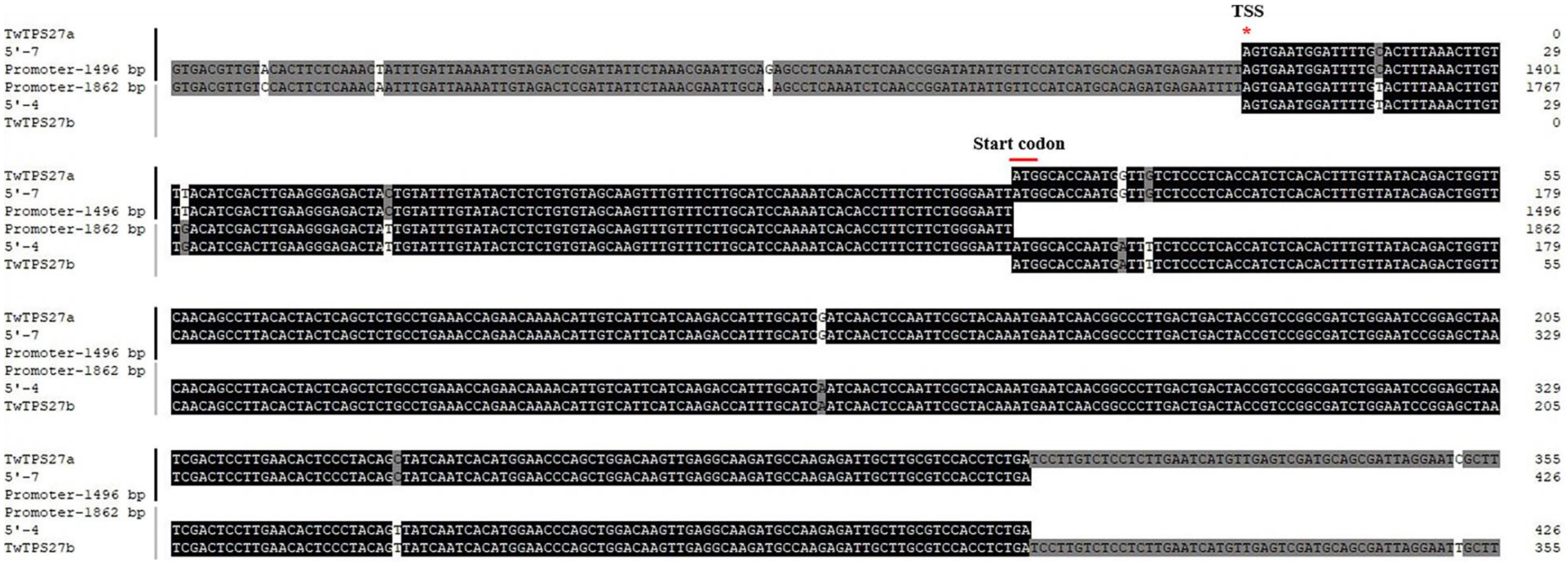

Figure S7. Sequence alignment for the determination of the *TwTPS27a* and *TwTPS27b* promoters. The 5'-7 and 5'-4 represent the 5'-RACE sequences of *TwTPS27a* and *TwTPS27b* from 5'-RACE assays, respectively. The TwTPS27a and TwTPS27b represent the coding sequences of *TwTPS27a* and *TwTPS27b*, respectively. The red asterisk represents the transcription start site (TSS). The start codons (ATGs) of *TwTPS27a/b* are marked with a red sigle line. After sequences alignment, the 1496 bp promoter sequence belongs to the *TwTPS27a* gene, whereas the 1862 bp promoter sequence belongs to the *TwTPS27b* gene.

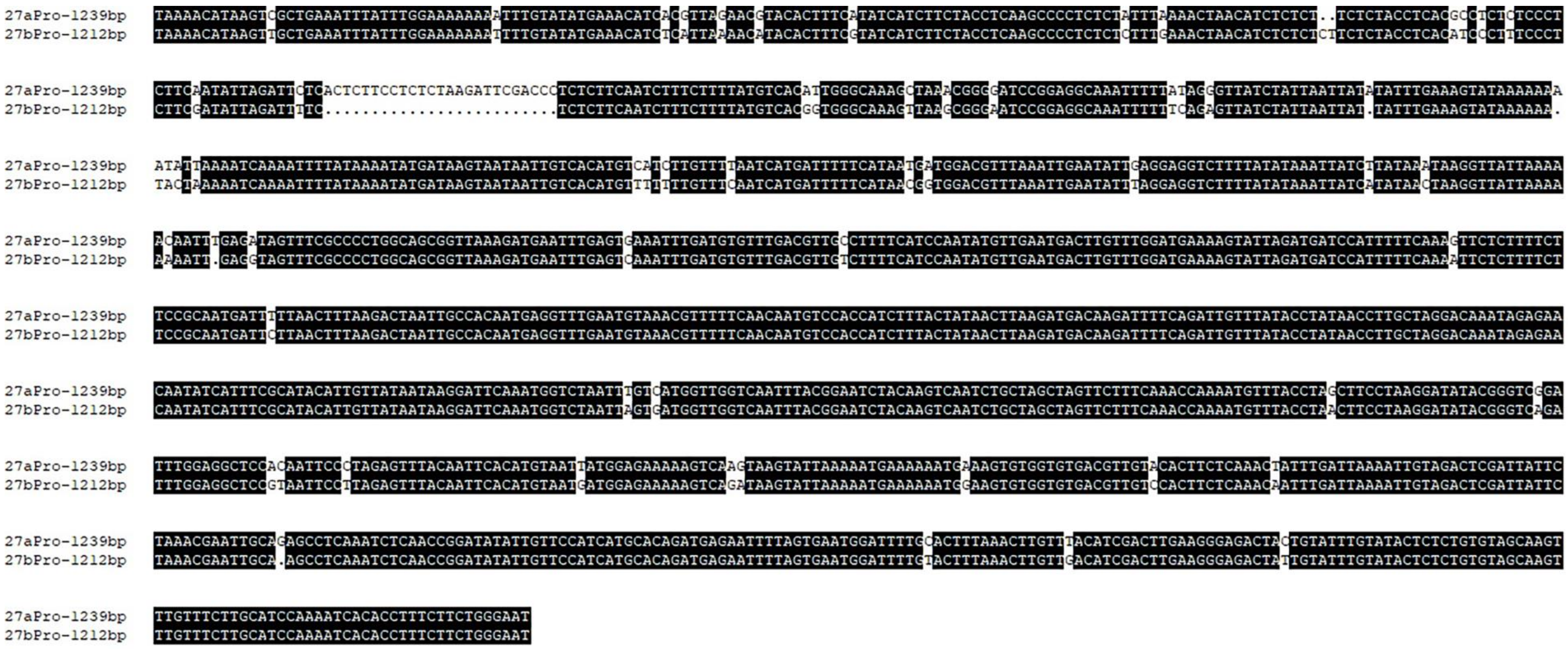

Figure S8. Sequence alignment between *TwTPS27a* promoter from −1115 to +124 bp and *TwTPS27b* promoter from −1088 to +124 bp. The two sequences showed 93.0 % nucleotide identity.

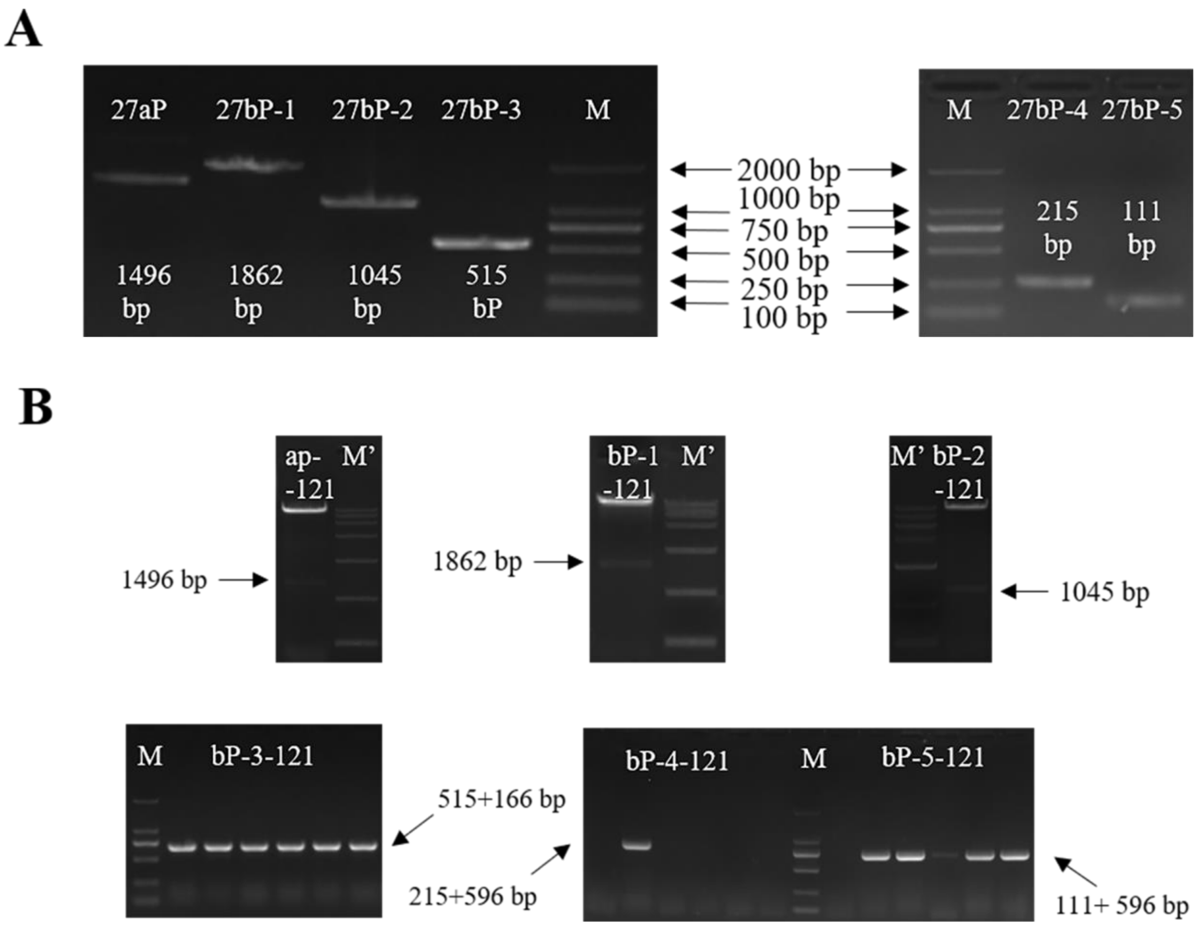

Figure S9. Verification of various promoter::GUS vectors. The vectors of aP-121, bP-1-121 and bP-2-121 were verified by double digestion with the restriction enzymes of *Hin*d III and *Xba* I. The vector of bP-3-121 was verified by the primers of M13R-4921: RCAGGAAACAGCTATGACC and 121-GUS-R: CAACGCTGATCAATTCCACAG. The vectors of bP-4-121 and bP-5-121 were verified by the primers of 121-cx-F4703：TCGCCCTTTTGTCTTTGGCCCAATAC and 121-cx-R6157：TCACTTCCTGATTATTGACCCACACTTTG.

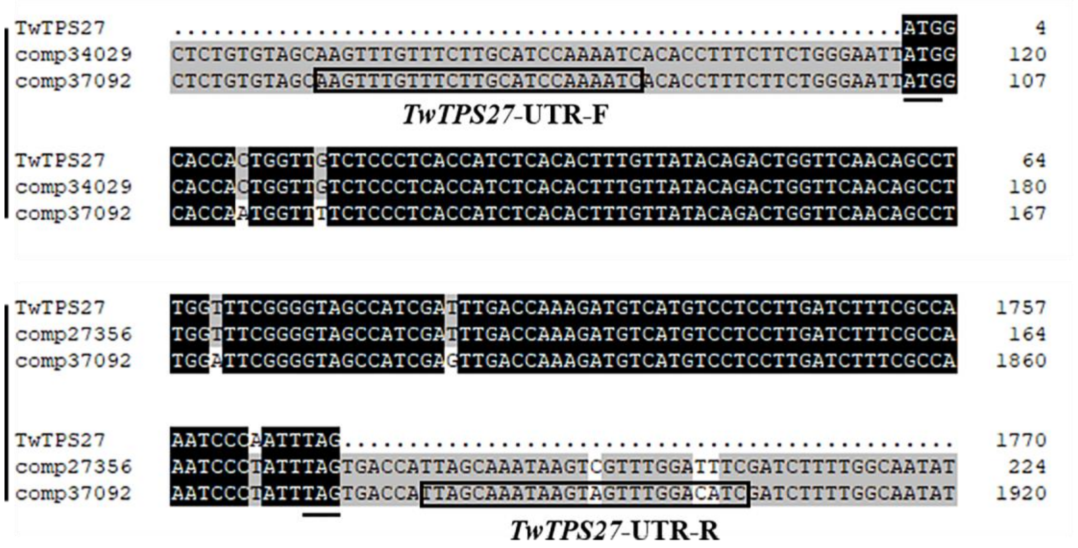

Figure S10. Primers designed for cloning the CDSs and gDNA sequences of *TwTPS27a/b*. The primers were designed in the 5'/3'-untranslated region (5'/3'-UTR) of the candidate genes. Single line represents the start/stop codons. The black boxes represent the sense and antisense primers.
